# Inhibition of Th17 cells by donepezil ameliorates experimental lung fibrosis and pulmonary hypertension

**DOI:** 10.7150/thno.82069

**Published:** 2023-03-21

**Authors:** Yuan Guo, Ziyu He, Zhu Chen, Fengling Chen, Chengming Wang, Wanlu Zhou, Jie Liu, Hao Liu, Ruizheng Shi

**Affiliations:** 1 Department of Cardiovascular Medicine, Xiangya Hospital, Central South University, Changsha 410008, Hunan, China.; 2 Department of Cardiovascular Medicine, Zhuzhou Hospital Affiliated to Xiangya School of Medicine, Central South University, Zhuzhou 412007, Hunan, China.; 3 Hunan Key Laboratory of Biomedical Nanomaterials and Devices, Hunan University of Technology, Zhuzhou 412007, Hunan, China.

**Keywords:** pulmonary hypertension, fibrosis, inflammatory response, T helper 17 cells

## Abstract

**Rationale:** Pulmonary hypertension (PH) secondary to lung fibrosis belongs to WHO Group III, one of the most common subgroups of PH; however, it lacks effective treatment options. Cholinesterase inhibitor donepezil (DON) has been shown to effectively improve Group I PH. However, its effects on Group III PH are unknown.

**Methods:** A lung fibrosis-induced PH mouse model was constructed using a single intratracheal instillation of bleomycin (BLM), after which DON was administered daily. Pulmonary artery and right ventricle (RV) remodeling were evaluated at the end of the study. Lung tissue in each group was analyzed using RNA sequencing, and the results were further verified with datasets from patients with PH. The mechanisms underlying DON-induced effects on PH were verified both *in vivo* and* in vitro*.

**Results:** DON effectively improved pulmonary artery and RV remodeling in the BLM-induced mouse model. Transcriptomic profiles of lung tissue indicated that the expression of inflammatory and fibrotic genes was significantly changed in this process. In the animal model and patients with PH, T helper 17 lymphocytes (Th17) were the most common inflammatory cells infiltrating the lung tissue. DON significantly inhibited lung fibroblast activation; thus, preventing lung fibrosis and reducing the inflammatory response and Th17 cell infiltration in the BLM-induced lung tissue. In addition, Th17 cells could activate lung fibroblasts by secreting IL17A, and DON-mediated inhibition of Th17 cell differentiation was found to depend on the α7nAchR-JAK2-STAT3 pathway.

**Conclusion:** DON can alleviate lung fibrosis and PH in an experimental mouse model. It inhibited pro-inflammatory Th17 cell differentiation, which is dependent on a cholinergic receptor pathway, thereby regulating fibroblast activation.

## Introduction

Pulmonary hypertension (PH) is a progressive malignant disease characterized by remodeling of small pulmonary vessels due to excessive inflammatory response and arteriole constriction 1. The etiology and pathology of PH are complicated and could be triggered by various factors, resulting in high heterogeneity in the classification, treatment, and prognosis 1, 2. Group III PH is secondary to pulmonary diseases, while lung fibrosis is the most common interstitial pulmonary disease, which, typically, indicates a high incidence of PH and portends a poor prognosis 3. PH secondary to lung fibrosis lacks sufficient therapeutic options, and the initial therapeutic strategy has shown limited results 3, 4. Therefore, urgent research is needed to explore novel therapeutic approaches to treat lung fibrosis-induced PH.

Lung fibrosis is an end-stage pulmonary disease characterized by fibroblast activation and excessive extracellular matrix deposition, leading to obliteration of the original tissue architecture and function 5-7. Although fibrosis is the final common pathway for various diseases, effective approach to prevent or counteract it is lacking; this makes lung fibrosis-induced PH refractory to treatment 5, 8. Usually, the development of fibrosis is related to the interplay between immune cells and fibroblast stimulation 9. This suggests immunotherapies might be a breakthrough for fibrotic disorder treatment.

Evidence demonstrates that parasympathetic activation exerts key cardiovascular protection via the “cholinergic anti-inflammatory pathway,” which mainly refers to innate immunity 10, 11. Both our and other studies reported that parasympathetic activation markedly reduced pulmonary arterial pressure and improved right heart function in Group I PH animal models, which is primarily dependent on its potent anti-inflammatory function via inhibiting macrophage 12-14. Thus, the anti-inflammatory role of parasympathetic activation has been regarded as the intermediate hub of neuroimmune regulation 10.

Interestingly, recent studies have reported the distribution of inflammatory cells in the lung tissue in patients with idiopathic PH and demonstrated that besides macrophages, other kinds of immune cells, especially T lymphocytes, play an essential role in the progress of PH secondary to lung fibrosis 15, 16. However, the regulatory function of parasympathetic activation on T lymphocytes is less reported. Whether parasympathetic activation exerts anti-inflammatory effects on lung fibrosis-induced PH and its regulation of T lymphocyte differentiation have not been determined.

In this study, an acetylcholinesterase (AchE) inhibitor, donepezil (DON), was used to treat the bleomycin (BLM)-induced PH mouse model. Transcriptomic profiles of lung tissue showed that the expression levels of genes regulating inflammatory and fibrotic processes were most significantly altered. Data from patients with PH verified the presence of T helper 17 (Th17) cells in lung tissue; subsequently, the underlying mechanisms of DON regulating Th17 cell differentiation, thereby mediating fibroblast activation, were analyzed. Overall, this study aimed to provide evidence and potential immunotherapy for lung fibrosis-induced PH from the perspective of neuromodulation.

## Materials and Methods

### Animals

**Figure 1:**
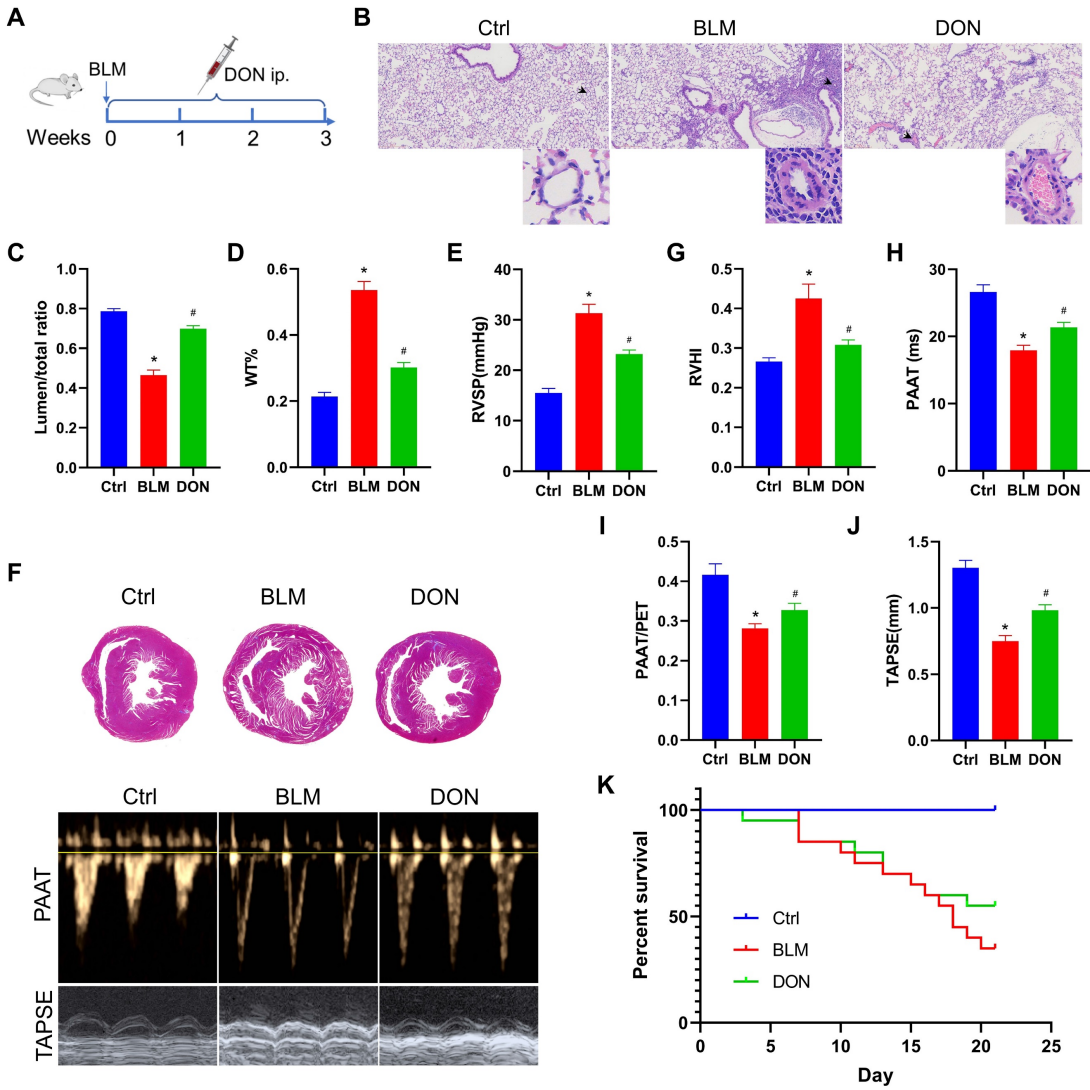
DON ameliorates BLM-induced pulmonary arterial and RV remodeling. A. Study design and scheme timeline. B. Representative images of HE-stained lung sections under a light microscope for each group; the arrow points to pulmonary vessels, and the enlarged vessel is presented in the bottom-right corner (×400 magnification, image size: 715 × 408 μm). C. Statistical analysis of the pulmonary arterial lumen to vessel diameter (n = 6). D. Statistical analysis of the pulmonary arterial WT% (n = 6). E. Statistical analysis of the RVSP (n = 8). F. Representative Masson's trichrome staining of heart samples and echocardiography, respectively, in the parasternal short axis and apical four chamber heart view. G. Quantitative analysis of RVHI (n = 7). H. Statistical analysis of PAAT (n = 7). I: Statistical analysis of PAAT/PET (n = 7). J: Statistical analysis of TAPSE (n = 7). K: Statistical analysis of the survival rate (n = 20). BLM: bleomycin, Ctrl: control, DON: donepezil, HE: hematoxylin and eosin, LV: left ventricle, PAAT: pulmonary arterial acceleration time, PET: pulmonary ejection time, RV: right ventricle, RVHI: RV hypertrophy index, RVSP: right ventricular systolic pressure, S: septum, TAPSE: tricuspid annular plane systolic excursion. WT: wall thickness. *P < 0.05, *versus* Ctrl group, ^#^*P* < 0.05, *versus* BLM group.

Susceptible male C57BL/6 mice (8 weeks old) were randomly divided into three groups: control (Ctrl), BLM, and bleomycin plus DON groups (n = 16 in each group). To construct a lung fibrosis-induced PH mouse model, a single dose of 2 mg/kg BLM (Selleck, Houston, TX, USA) was administered intratracheally on day 1 of the experimental procedure. To determine the survival rate, a single dose of 5 mg/kg bleomycin was used (n = 20 in each group). An equal volume of saline was administered in the control group. DON (1 mg/kg, Selleck) was intraperitoneally injected daily from the day 1 to day 21 as a therapeutic protocol (Figure 1A). At the end of the study, samples were collected from animals after anesthesia with pentobarbital injection (180 mg/kg). The Animal Research Committee of Central South University in Hunan, China, approved all animal care and experiments (Approval No. 2020SYDW0986).

### Datasets collection and analysis for patients with PH

Datasets from Gene Expression Omnibus (GEO) were systematically searched to explore the molecular mechanism of PH secondary to lung fibrosis. Three microarray datasets (GSE24988, GSE48149, and GSE15197) related to Homo sapiens and containing both patients with PH related to lung fibrosis and non-PH controls were obtained and analyzed. The dataset information and analysis procedures are presented in Figure S1 and Table S1.

Briefly, series matrix file(s) and corresponding annotation documents of the three datasets were downloaded from the GEO website. Data preprocessing, conversion, and missing value recovery were conducted on the Sangerbox platform 17. Next, the “InSilicoMerging” packages in R were used to combine the three datasets, and Empirical Bayes was used to batch normalize, thereby obtaining co-expression genes of the three datasets. Integrated gene expression data from different datasets were confirmed in an unbiased manner (Figure S2). Further, linear models for microarray data (Limma) analysis were used to obtain statistically changed genes between the non-PH group and the PH group, and the threshold of differentially expressed genes (DEGs) was set at *P* < 0.05 and |log2FC| > 1.5. The functional enrichment analysis of DEGs was conducted using the *Database for Annotation, Visualization, and Integrated Discovery (DAVID) tool*. Finally, gene ontology (GO) classification terms (BP, biological process) of DEGs were searched and visualized.

In addition, Gene Set Enrichment Analysis (GSEA) was used to explore potential biological mechanisms in which *P* < 0.05 or Benjamini-Hochberg's false discovery rate (FDR) < 0.25 indicated significant differences. Moreover, the immune cell landscape in lung tissue was identified via *ImmuCellAI biotools*. Lastly, the least absolute shrinkage and selection operator (LASSO) logistic regression was used to verify the signature of the most significantly changed immune cells in the PH process.

### Isolation and cultivation of mouse lung fibroblasts

Lung fibroblasts were isolated and cultured according to a modified proven protocol 18. Briefly, intraperitoneally anesthetized mice with pentobarbital were thoroughly sterilized with 75% ethanol. Next, the lung tissue was separated, isolated and cut into tiny pieces using sterile forceps and scissors. Lung fragments were digested with 2 mg/mL collagenase type II (Worthington Biochemical, USA) for 30 minutes at 37 ℃. After being purified by adhesion for 40 minutes, lung fibroblasts were cultured in Dulbecco's Modified Eagle Medium (DMEM) medium containing 10% fetal bovine serum (FBS, Gibco, USA) and 1% penicillin-streptomycin (Gibco, USA). Lung fibroblasts were determined as stain positive with vimentin after 6 days of cultivation (Figure S3).

### *In vitro* co-culture of lung fibroblasts and Th17 cells

Lung fibroblasts were distributed in 6-well plates at a concentration of 5×10^5^ cells/well. After incubating for five hours, half the medium was removed, and 1×10^5^ Th17 cells were added into each well and co-cultured at 37 °C with 5% CO_2_. Besides, 1 µg/mL anti-IL17A (R&D Systems, USA) neutralizing antibody was added to the lung fibroblasts 1 hour before adding Th17 cells. Subsequently, the co-culture was performed with or without the anti-IL17A antibody. The activity of lung fibroblasts was tested after 48 hours co-incubation.

### Other procedures and data availability

Measurement of echocardiography and assessment of hemodynamics, histology of lung and heart, RNA sequencing (RNA-seq), flow cytometry, T lymphocyte isolation and Th17 cell differentiation, immunohistochemical staining, immunofluorescence staining, mouse inflammation antibody array detection, ELISA test, western blot, and real-time quantitative PCR (RT-PCR) are available in the supplemental material and methods. RNA-seq data of mice have been deposited in the Gene Expression Omnibus (GEO) under the accession code GSE211224. Patient data were downloaded from publicly available datasets, which are available in the public GEO database (Accession numbers: GSE24988, GSE48149, and GSE15197). All data and methods relevant to the findings of this study are available from the corresponding authors at request.

### Statistical analysis

All non-sequencing data statistical analysis was performed in GraphPad Prism software (v.8, San Diego, CA, USA). Data are presented as mean ± standard error of the mean (SEM). Multi-group comparison data were subjected to one-way ANOVA while Tukey's multiple comparisons were used to determine the statistical significance of the simple effect between groups. The difference between the two groups was compared using a paired t-test. A *P* < 0.05 was considered statistically significant.

## Results

### DON effectively improves cardiopulmonary function in a BLM-induced mouse model

To determine the effects of DON on pulmonary arterial remodeling in BLM-induced mice, the lumen stenosis and thickness of the pulmonary artery were measured using light microscopy. Histological analyses of pulmonary arterioles showed a decrease in the lumen diameter ratio from 78.7% in the control group to 46.4% in the BLM group, which increased to 69.8% after DON treatment; however, the wall thickness (WT)% of the pulmonary artery showed the opposite result (all *P* < 0.05, Figure 1B-D). This suggests that DON could significantly reverse pulmonary arterial remodeling in lung fibrosis-induced PH. Interestingly, the remodeling of the muscular pulmonary arteries and arterioles was more likely to be restricted to the fibrotic lung areas, indicating that this improvement is based on alleviating the fibrotic process.

The RV systolic pressure (RVSP) was measured using a close-chest method to evaluate the right heart remodeling. In the BLM-induced model mice, the RVSP increased to 31.3 mmHg, which is markedly higher than that in the control group, and decreased significantly to 23.2 mmHg after DON treatment (*P* < 0.05, Figure 1E). In addition, the right ventricle hypertrophy index, another parameter that represents right heart hypertrophy and remodeling, was markedly increased in BLM-induced mice compared with that in the control group; however, it was effectively reversed after DON treatment (*P* < 0.05, Figure 1F-G). These results showed that DON improved RV remodeling in the experimental lung fibrosis-induced PH mouse model.

Echocardiography was used to measure the RV function. The mean values of pulmonary arterial acceleration time (PAAT) and PAAT/pulmonary ejection time (PET) decreased from 26.6 ms and 41.6%, respectively, in the control group to 17.9 ms and 28.2%, respectively, in the BLM group. In comparison, they increased to 21.4 ms (PAAT) and 32.8% (PAAT/PET) after DON treatment (all *P* < 0.05, Figure 1F and H-I). Additionally, TAPSE, an indicator that reflects RV contractile function, decreased significantly in the BLM-induced mice, and this decrease was also reversed after DON treatment (all *P* < 0.05, Figure 1J). These indicators showed that DON effectively reversed RV remodeling and dysfunction in the BLM-induced mouse model, reflecting the change in pulmonary arterial pressure.

Accordingly, a higher dosage of BLM was used to evaluate the survival rate of mice after DON treatment. The survival rate of mice increased to 55% in the DON group compared with the 35% survival in the BLM group (Figure 1K), which further reflects the improvement in cardiopulmonary function after DON intervention.

### DON mainly improved fibrotic and inflammatory processes in PH secondary to lung fibrosis

**Figure 2:**
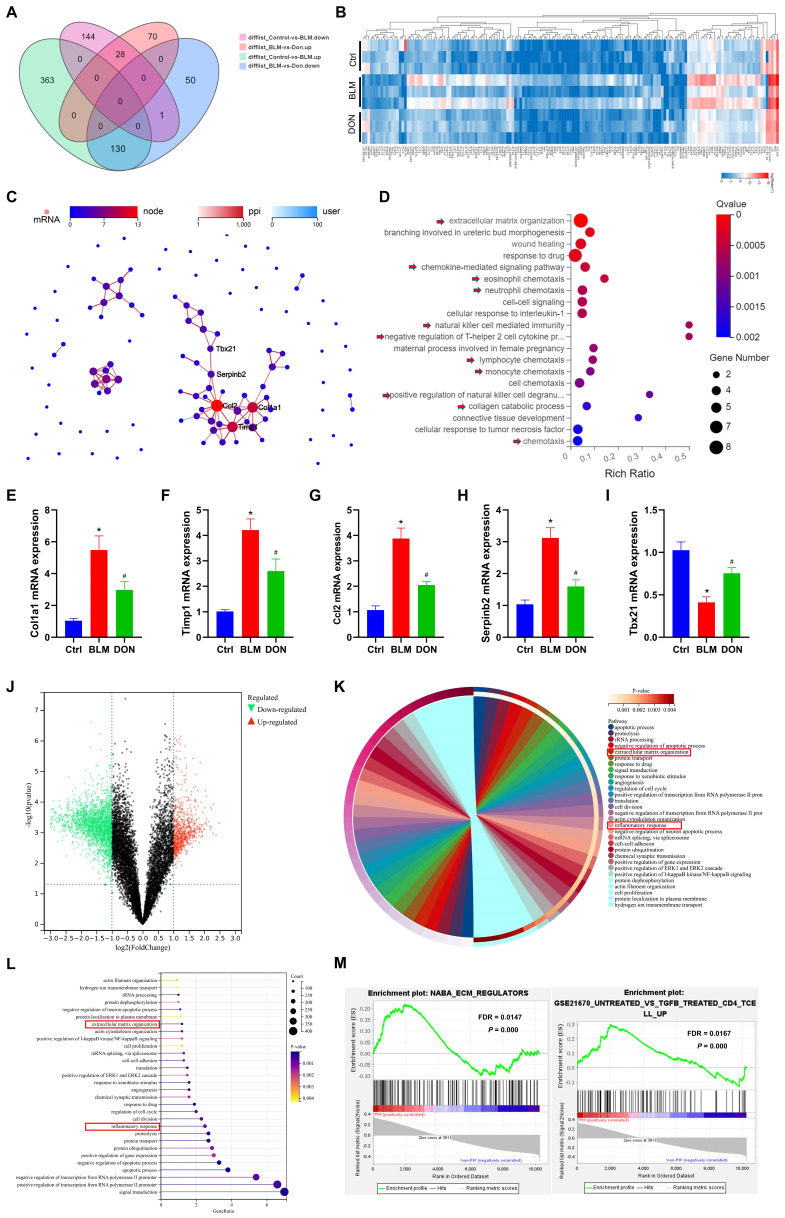
DON improved fibrotic and inflammatory effects in lung fibrotic process. A. Venn diagram of the DEGs in each group identified by RNA-seq. B. Heat map of RNA-seq expression data showing 159 genes that were differentially regulated in BLM‑induced mice after DON treated (n = 3). C. PPI network of DEGs, among which, *Col1a1*, *Timp1*, *Ccl2*, *Serpinb2*, and *Tbx21* were the most closely connected. D. Bubble plot of GO analysis for the 40 functional most connected genes in the PPI network. E~I: Statistical analysis of the mRNA expression of *Col1a1*, *Timp1*, *Ccl2*, *Serpinb2*, and *Tbx21* in lung tissue. J. volcano plot to show the DEGs between patients with PH secondary to lung fibrosis (n = 78) and non-PH controls (n = 44). K. Chord plot to show GO-enriched items of DEGs. L. Lollipop graph to show GO-enriched items of DEGs. M. The GSEA analysis revealing the enriched pathways in the patients with PH and control samples. Ctrl: control, BLM: bleomycin, DON: donepezil. DEGs: differentially expressed genes, GO: gene ontology, GSEA: gene set enrichment analysis, PPI: protein-protein interaction. *P < 0.05, *versus* Ctrl group, ^#^*P* < 0.05, *versus* BLM group.

To explore the underlying mechanism of DON in BLM-induced mice, lung tissue was subjected to RNA-seq analysis. Compared with the control group, 493 genes were upregulated whereas 173 were downregulated in the BLM group. Meanwhile, compared with DON-treated mice, 98 genes were upregulated and 181 were downregulated in BLM-induced mice (Figure 2A).

Thus, 159 genes were identified as DEGs in lung tissue that changed with the most significant difference among the three groups (Figure 2A-B, Table S2). To explore the potential function of these DEGs, protein-protein interaction (PPI) was further analyzed, and 40 DEGs were identified as most closely interacting, which were mainly associated with extracellular matrix organization and inflammatory regulation, such as chemokine-mediated signaling pathway, eosinophil chemotaxis, neutrophil chemotaxis, and natural killer cell-mediated immunity (Figure 2C-D, Figure S4).

To further confirm the effect of DON on the BLM-induced mice, five genes in the core of the PPI network, *Col1a1* (encoding collagen type I alpha 1 chain), *Timp1* (encoding TIMP metallopeptidase inhibitor 1), *Ccl2* (encoding C-C motif chemokine ligand 2), *Serpinb2* (encoding serpin family B member 2), and *Tbx21* (encoding T-box transcription Factor 21) were quantitatively analyzed in lung tissue. Compared with the control group, *Col1a1, Timp1, Ccl2*, and *Serpinb2* expression levels were increased to 5.5-fold, 4.2-fold, 3.9-fold, and 3.1-fold, respectively, while that of *Tbx21* decreased to 2.5-fold in the BLM group (all *P* < 0.05, Figure 2E-I). However, after DON administration, the relative mRNA expression levels of *Col1a1*,* Timp1*,* Ccl2*, and *Serpinb2* were significantly decreased, while that of *Tbx21* increased compared with their corresponding expression levels in the BLM group (all *P* < 0.05, Figure 2E-I). These results validated that DON could effectively reverse the fibrotic and inflammatory process in lung fibrosis in a PH animal model.

Next, datasets involving patients with PH secondary to lung fibrosis were analyzed to further confirm this effect in humans. A total of 5738 overlapping DEGs, including 1791 upregulated and 3947 downregulated genes, were identified in patients with PH secondary to lung fibrosis compared to non-PH controls (Figure 2J). Further, GO visualized the biological processes of the DEGs and drew a similar conclusion in patients with PH (Figure 2K-L). Consistently, GSEA analysis results also indicated that extracellular matrix regulation and inflammatory processes, especially the T lymphocyte-induced immune response, were significantly changed in patients with PH secondary to lung fibrosis (Figure 2M, all *P* < 0.05 and FDR < 0.25), indicating that these pathological processes in animal model accurately simulated the biological effects of patients with PH.

### DON inhibits lung fibrosis in BLM-induced mice by reducing fibroblast activation

**Figure 3:**
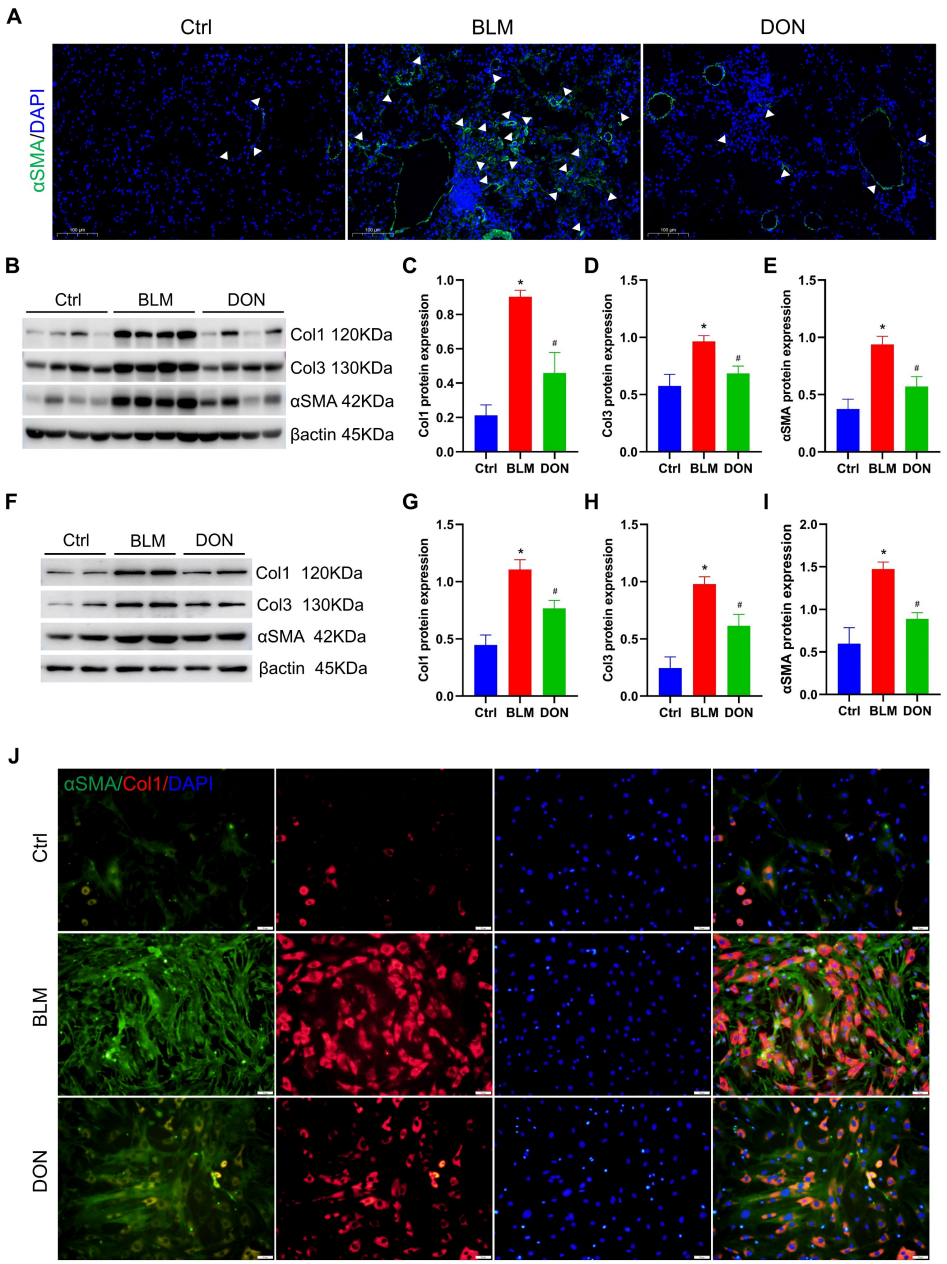
DON ameliorates lung fibrosis in BLM-induced mice by inhibiting fibroblast activation. A. Representative immunofluorescent staining with αSMA for lung tissue (×200 magnification). B. Representative protein levels in lung tissue. C~E. Statistical analysis of the levels of collagen I, collagen III, and αSMA in lung tissue (n = 8). F. Representative protein levels in lung fibroblasts. G~I. Quantitative analysis of the levels of collagen I, collagen III, and αSMA in lung fibroblasts (n = 4). J: Representative immunofluorescent staining with collagen I and αSMA for fibroblasts (×200 magnification). αSMA: alpha-smooth muscle actin, BLM: bleomycin, Ctrl: control, Col1: collagen I, Col3: collagen III, DON: donepezil. *P < 0.05, *versus* Ctrl group, ^#^*P* < 0.05, *versus* BLM group.

Immunofluorescence staining for αSMA was employed to assess the activation of lung fibroblasts *in situ*. Notably, there was a significant increase in αSMA-positive staining in lung tissue from mice treated with BLM, which was effectively attenuated by treatment with DON (Figure 3A). The protein levels of collagen I, collagen III, and αSMA in lung tissue were measured for quantitative analysis of the fibrosis. When compared with the mice in the control group, the relative levels of collagen I, collagen III, and αSMA were increased in the BLM-induced mice (4.2-fold, 1.7-fold, and 2.5-fold, respectively); while their expression levels were prominently decreased in the DON treated group (all *P* < 0.05, Figure 3B-E). This verified that DON could inhibit lung fibrosis in the BLM-induced mouse model and subsequently offered a basis for improved pulmonary arterial and RV remodeling.

Besides, lung fibroblasts were isolated from each group and cultured *in vitro* to explore the underlying mechanism. After 6 days of cultivation, the levels of collagen I, collagen III, and αSMA were further assayed to evaluate their activation. Consistently, the levels of collagen I, collagen III, and αSMA were increased significantly in the BLM group as compared with those in the control group, and DON effectively reversed this change (all *P* < 0.05, Figure 3F-I). Immunofluorescence staining for collagen I and αSMA was performed to test this further. As shown in Figure 3J, the positive rate of collagen I and αSMA in lung fibroblasts was significantly increased in the BLM group and markedly reduced in the DON group (Figure 3J). Therefore, DON could effectively inhibit the activation of lung fibroblasts, which directly contributed to alleviating the fibrotic process in BLM-induced mice.

### DON reduces the Th17-mediated inflammatory response in the BLM-induced mouse model

**Figure 4:**
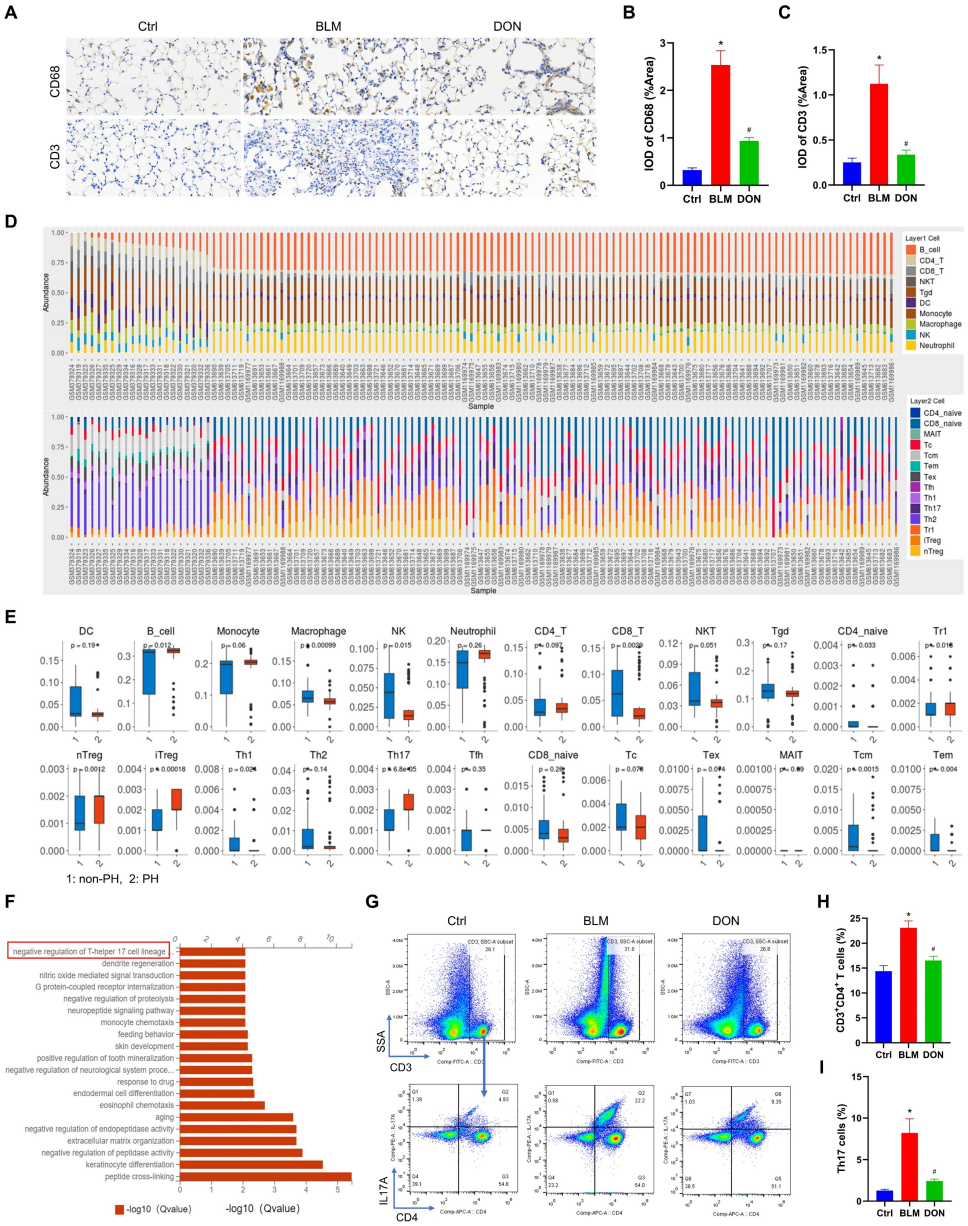
DON reduces the Th17-mediated inflammatory response in BLM‑induced mice model. A. Representative immunohistochemical staining for CD68 and CD3 (×400 magnification, image size: 715 × 408 μm). B~C. Quantitative analysis of the CD68 and CD3 positive rate. D. The abundance of 24 types of immune cell infiltrating in lung tissue of non-PH and PH patients. E. Boxplot of the proportion of 24 types of immune cells. F. GO analysis indicating the major significant biological processes, includes negative regulation of Th17 cell lineage commitment. G. The original flow cytometry plots. H. Quantitative analysis the percentage of CD4^+^ T lymphocytes in lung tissue. I. Quantitative analysis the percentage of Th17 cells in lung tissue. Ctrl: control, BLM: bleomycin, DON: donepezil, GO: gene ontology, IL17A: interleukin 17A, Th17: T helper 17. *P < 0.05, *versus* Ctrl group, ^#^*P* < 0.05, *versus* BLM group, (n = 6).

To verify the infiltration of inflammatory cells in lung tissue, immunohistochemical staining with CD68 and CD3 was performed. Immunohistochemistry analysis showed that the CD68 and CD3 stained IOD/area increased from 0.32 and 0.25 in the control group to 2.53 and 1.12 in the BLM group and decreased to 0.94 and 0.34 in the DON group, respectively (all *P* < 0.05, Figure 4A-C). This result showed that DON could effectively reduce the inflammatory infiltrate in BLM-induced lung tissue.

To observe the immune cell landscape in corresponding patients, we further analyzed 24 types of immune cell infiltration in lung tissue. The results showed a significant cluster of immune cells infiltrated the lung tissue, including B cells, macrophages, NK cells, and T cells subtypes (all *P* < 0.05, Figure 4D-E). Among these, the change in Th17 cells was the most significant (*P* < 0.0001, Figure 4E). The correlation and stark difference between the 24 different types of immune cells infiltrating the lung tissue of patients with PH and the paucity of inflammatory cells in the non-PH controls is shown in Figure S5. LASSO logistic regression was used to detect the signature of the Th17 cells in the PH course, and the model was constructed with DEGs, from which its risk score correlated with Th17 cells (Figure S6). This validated the essential function of Th17 cells in the PH course.

The function of DEGs from mice was also analyzed to investigate the underlying mechanism of DON in regulating the BLM-induced mouse model. GO analysis revealed the top 20 correlated functional pathways of DEGs from the three groups, also including negative regulation of Th17 cell lineage commitment (Figure 4F). Th17 cells are the most widely studied pro-inflammatory cells, which possess essential profibrotic properties. This suggested that DON improvement of BLM-induced lung fibrosis might be dependent on inhibiting Th17 cell differentiation.

To further elucidate the infiltration of Th17 cells in lung tissue, flow cytometry detection of CD4^+^ T lymphocytes and Th17 (CD3^+^CD4^+^IL17A^+^ T) cells was performed. Compared with that in the control group, the number of CD4^+^ T lymphocytes and Th17 cells was increased in the BLM group (CD4^+^ T lymphocytes: 14.38± 2.74 *vs.* 23.07 ± 3.43, Th17 cells: 1.28 ± 0.37 *vs.* 8.21 ± 4.19, respectively), while both decreased significantly after DON treatment (all *P* < 0.05, Figure 4G-I, Figure S7). This finding confirmed that DON effectively inhibited Th17 cell activation, which might be the main reason for alleviating the fibrotic process in PH.

### DON regulates Th17 cell differentiation *in vitro*

**Figure 5:**
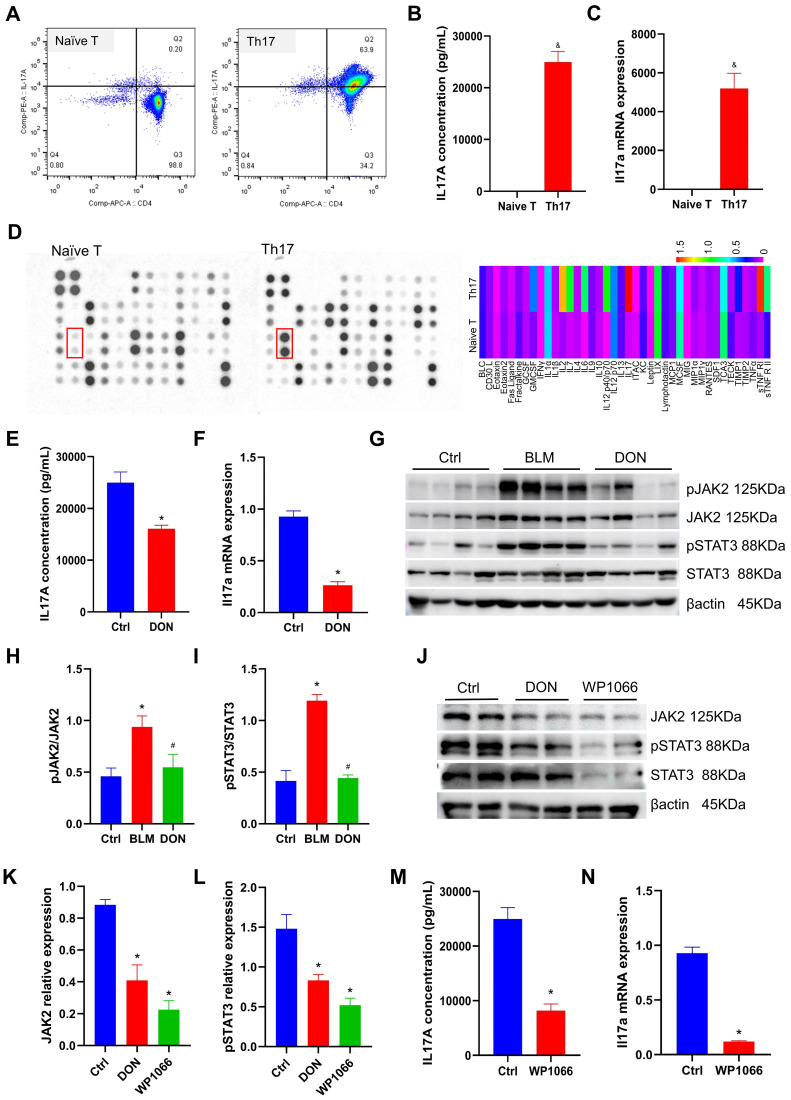
DON regulates Th17 differentiation by inhibiting JAK2-STAT3 pathway. A. Flow cytometry to detect naïve T lymphocytes differentiating into Th17 cells. B. Quantitative analysis of IL17A levels in Th17 differentiation medium (n = 5). C. Quantitative analysis of *Il17a* mRNA expression (n = 4). D. Mouse inflammation antibody array-membrane to detect inflammatory factors and cytokines in Th17 differentiation medium and heat map show the grayscale values. E. Quantitative analysis of IL17A levels after DON treatment (n = 5). F. Quantitative analysis of *Il17a* mRNA expression after DON treatment (n = 4). G. Representative protein levels in lung tissue. H~I. Statistical analysis of the levels of pJAK2 and pSTAT3 in lung tissue (n = 8). J. Representative protein levels in Th17 cells. K~L. Statistical analysis of the levels of JAK2 and pSTAT3 in Th17 cells (n = 4). M. Quantitative analysis of IL17A levels in Th17 cells after WP1066 treatment (n = 5). N. Quantitative analysis of *Il17a* mRNA expression in Th17 cells after WP1066 treatment (n = 4). Ctrl: control, BLM: bleomycin, DON: donepezil, IL17A: interleukin 17A, JAK2: Janus Kinase 2, STAT3: signal transducer and activator of transcription 3. Th17: T helper 17. *P < 0.05, *versus* Ctrl group, ^#^*P* < 0.05, *versus* BLM group, ^&^*P* < 0.05, *versus* Naïve T cells group.

To mimic the process of T lymphocytes differentiating into Th17 cells and to explore the underlying mechanism, naïve CD4^+^ T lymphocytes were isolated from mouse spleens and cultured with a Th17 differentiation medium. After 5 days of cultivation, the Th17 cell differentiation rate was assayed. As shown in Figure 5A, naïve CD4^+^ T lymphocytes were efficiently isolated and successfully differentiated into Th17 cells (Figure 5A). The cell morphology of naïve T lymphocytes and Th17 cells is shown in Figure S8. Moreover, the expression of IL17A was used to evaluate the activation of Th17 cells. The mean concentration of IL17A increased significantly to 24964.16 pg/mL in the Th17 culture medium (*P* < 0.05, Figure 5B). In addition, the mRNA expression of *Il17a* in Th17 cells also increased substantially (*P* < 0.05, Figure 5C). This suggests that Th17 cells were well differentiated.

To investigate the potential mechanism of Th17 cells, we employed a mouse inflammation antibody array-membrane to evaluate changes in inflammatory factors and cytokines during Th17 cell differentiation. A total of 40 mouse inflammatory factors and cytokines were measured, and it was found that 12 of these factors exhibited a greater than twofold change (Figure 5D and Table S3). Notably, IL17 showed the most significant increase in expression and was deemed to be the essential mediator for the functional activity of Th17 cells.

To test whether DON could regulate the process of naïve CD4^+^ T lymphocyte differentiation into Th17 cells, 5 μM DON was added to the conditioned medium on day 3. After another 48 hours of cultivation, the concentration of IL17A in the Th17 cell medium from the cells treated with DON decreased by 35.5% compared with that in the non-intervention group (*P* < 0.05, Figure 5E). Correspondingly, the mRNA expression of *Il17a* in Th17 cells decreased to 3.52-fold after DON intervention compared with that in the non-intervention group (*P* < 0.05, Figure 5F). This result indicates that DON could effectively hamper naïve CD4^+^ T lymphocyte differentiation into Th17 cells.

### JAK2-STAT3 pathway contributes to DON regulation of Th17 cell differentiation

In lung tissue, we detected that the JAK2-STAT3 pathway was significantly activated in the BLM-induced mouse model, which could be significantly inhibited by DON treatment. Furthermore, the relative levels of pJAK2 and pSTAT3 increased to 2.04-fold and 2.88-fold, respectively, in the BLM-induced mice compared with those in the control group; while they were substantially reduced after DON treatment (all *P* < 0.05, Figure 5G-I), suggesting the vital function of the JAK2-STAT3 pathway in the protective effect of DON.

Interestingly, the JAK2-STAT3 pathway is a key pathway that regulates T lymphocyte maturation and Th17 cell differentiation. Thus, we tested the changes in JAK2 and pSTAT3 levels in Th17 cells after DON treatment. Compared with those in the non-intervention group, the relative levels of JAK2 and pSTAT3 in Th17 cells decreased significantly after DON treatment (all *P* < 0.05, Figure 5J-L), indicating that DON regulation of Th17 cell differentiation is related to blockade of the JAK2-STAT3 signaling pathway.

The Th17 cell differentiation rate was further assayed after treatment with the specific inhibitor of JAK2, WP1066. At a dose of 2 μM, WP1066 significantly decreased JAK2 and pSTAT3 levels in Th17 cells (all *P* < 0.05, Figure 5J-L). After blocking this pathway, the concentration of IL17A in the Th17 cell medium was reduced to 8198.03 pg/mL (*P* < 0.05, Figure 5M), and the mRNA expression of *Il17a* in Th17 cells also decreased significantly (*P* < 0.05, Figure 5N), confirming that the JAK2-STAT3 signaling pathway could effectively determine the differentiation of Th17 cells.

### Th17 cell effectively stimulates lung fibroblast activation

**Figure 6:**
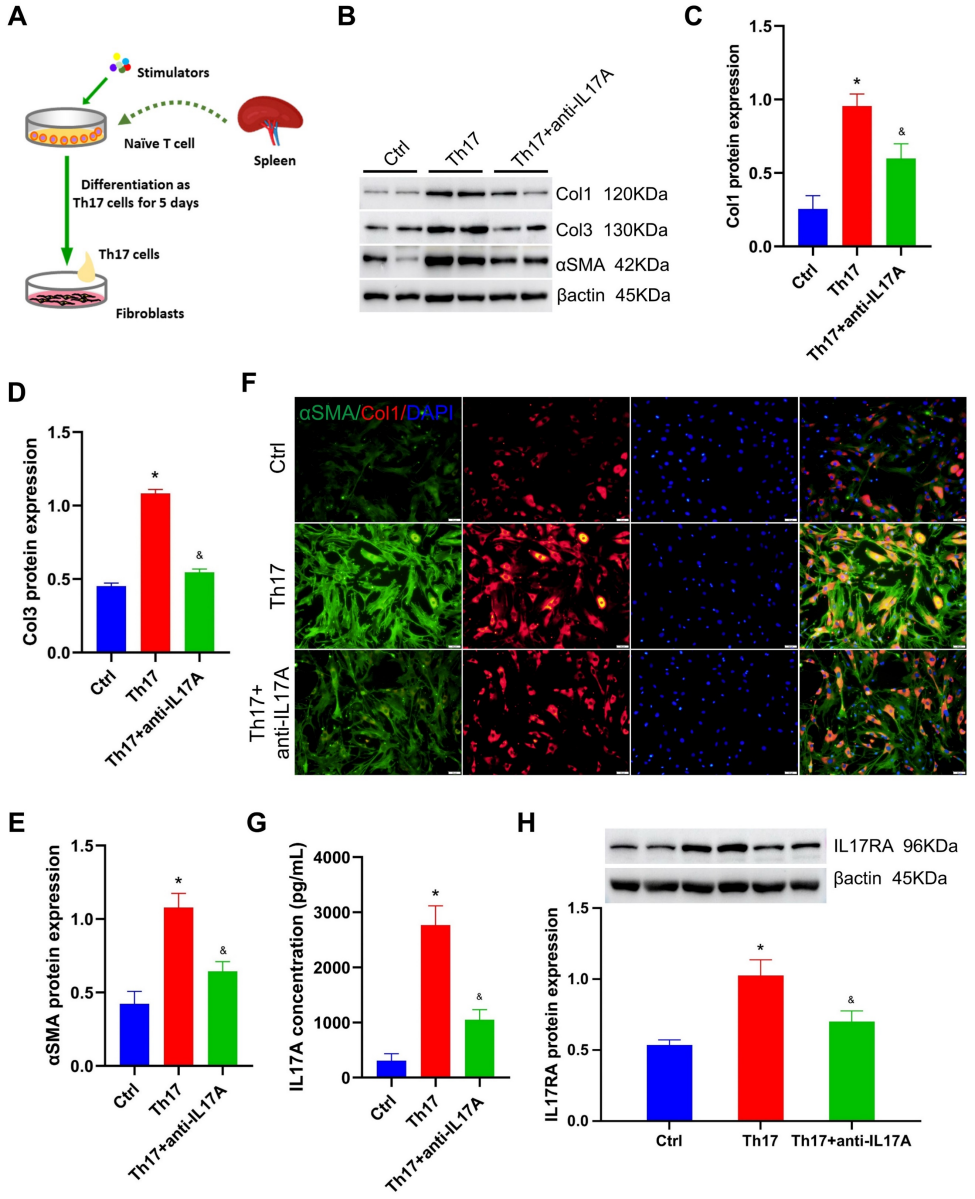
Th17 cell stimulates lung fibroblasts by secreting IL17A. A. Diagrammatic sketch of the co-cultivation of Th17 cells and lung fibroblasts. B. Representative protein levels in lung fibroblasts after co-cultivation. C~E. Statistical analysis of the expression of collagen I, collagen III, and αSMA in lung fibroblasts after co-cultivation (n = 4). F. Representative immunofluorescent staining for collagen I and αSMA (×200 magnification). G. Quantitative analysis of IL17A concentration in the co-culture medium (n = 5). H. The level of IL17RA in lung fibroblasts after co-cultivation (n = 4). αSMA: alpha-smooth muscle actin, Ctrl: control, Col1: collagen I, Col3: collagen III, IL17A: interleukin 17A, IL17RA: interleukin 17A receptor, Th17: T helper 17. *P < 0.05, *versus* Ctrl group, ^&^*P* < 0.05, *versus* Th17 cell group.

To test the effect of Th17 cells on lung fibroblasts, the fibroblast function was tested after co-culture with Th17 cells for 48 hours (Figure 6A). The relative protein levels of collagen I, collagen III, and αSMA increased significantly in the Th17 cell co-culture group compared with those in the control group (all *P* < 0.05, Figure 6B-E). Furthermore, immunofluorescence staining also revealed a significant increase in collagen I and αSMA levels in fibroblasts after co-culture with Th17 cells, compared with those in the control group (Figure 6F). This suggests that Th17 cells could significantly activate lung fibroblasts and promote the secretion of the extracellular matrix *in vitro*.

Generally, the Th17 cells' ability to exert their function depends on the secretion of inflammatory factors, especially IL17A. Therefore, neutralizing antibodies against IL17A, anti-IL17A, were used to exhaust IL17A during co-cultivation. Compared with those in the Th17 cell co-culture group, the relative levels of collagen I, collagen III, and αSMA decreased to 1.6-fold, 2.0-fold, and 1.7-fold, respectively, after exhausting IL17A using 1 μg/mL anti-IL17A (all *P* < 0.05, Figure 6B-E). Correspondingly, immunofluorescence staining also exhibited a significant reduction in collagen I and αSMA levels in fibroblasts treated with anti-IL17A (Figure 6F). This suggested that IL17A is the key regulator by which Th17 cells stimulate lung fibroblasts.

Besides, the IL17A concentration in the co-culture medium was assayed, which increased by 7.95-fold after Th17 cell treatment, and decreased by 62% after anti-IL17A treatment (all *P* < 0.05, Figure 6G). In addition, the level of the IL17A receptor (IL17RA) in fibroblasts was also detected. After co-culture with Th17 cells, the relative level of IL17A increased compared with that in the control group; however, its expression was significantly reduced after anti-IL17A treatment (all *P* < 0.05, Figure 6H). This result confirmed that Th17 cells activate lung fibroblasts by releasing inflammatory factor IL17A, which subsequently targets its receptor IL17RA to increase extracellular matrix secretion.

To further verify the effect of IL17A on fibroblasts, 0.1 μg/mL recombinant mouse IL17A protein (ABclonal, China) was treated with fibroblasts before IL17A was neutralized (or not). As shown in Figure S9, the relative levels of collagen I, collagen III, and αSMA were increased but not significantly after being treated with recombinant mouse IL17A protein, and anti-IL17A treatment was also not shown to significantly change the extracellular matrix secretion in fibroblasts (Figure S9A-D). This interesting result may indicate that Th17 cell-specific-derived IL17A exhibited potent profibrotic function in the PH process.

### DON regulates Th17 cell differentiation dependent on α7nAchR

**Figure 7:**
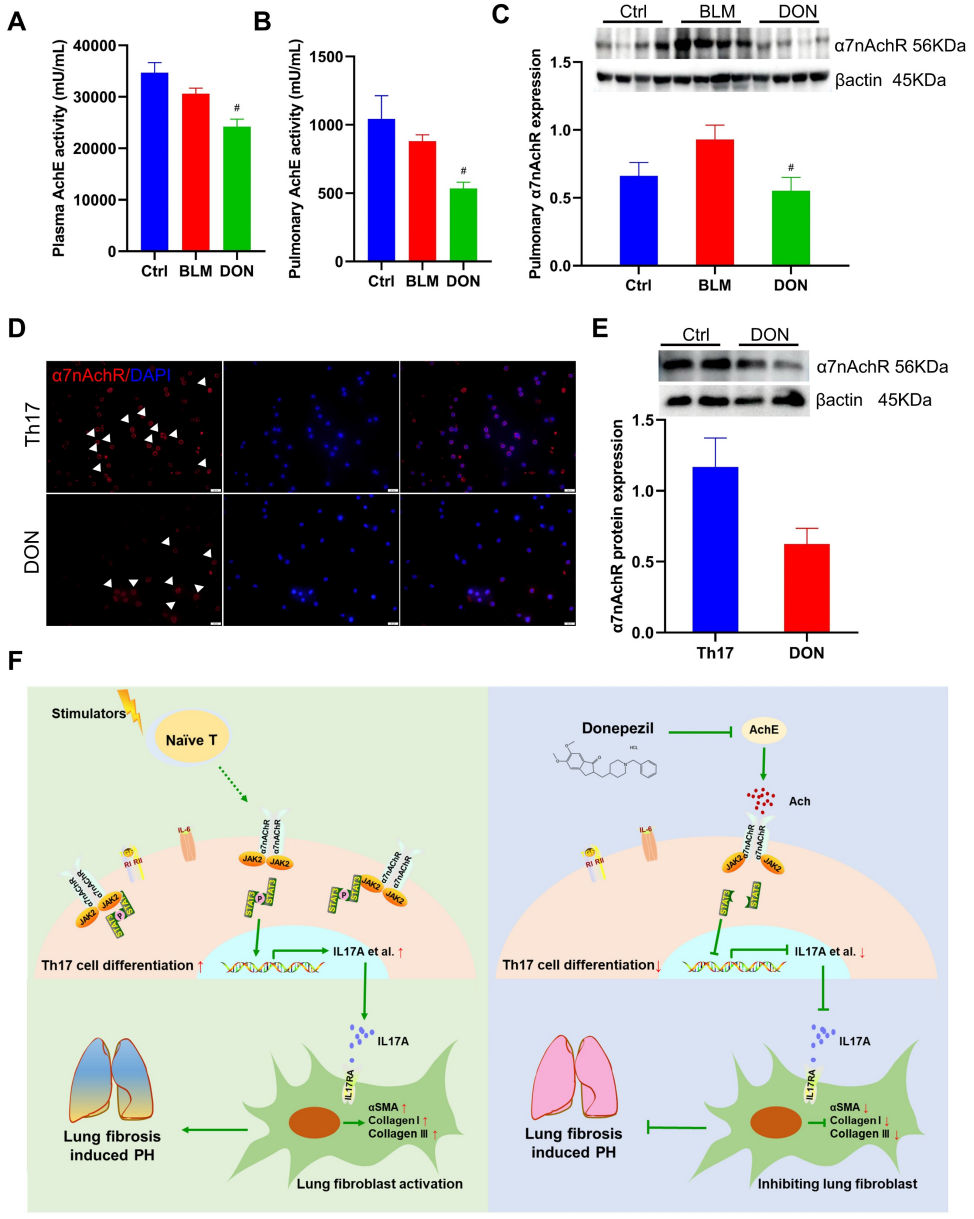
DON stimulates parasympathetic activation by inhibiting α7nAChR. A. Statistical analysis of AchE activity in plasma (n = 5). B. Statistical analysis of AchE activity in lung tissue (n = 5). C. Relative protein level of α7nAchR in lung tissue (n = 8). D. Representative immunofluorescent staining of α7nAchR on Th17 cells after DON treatment (×400 magnification). E. Relative protein level of α7nAchR in Th17 cells after DON treatment (n = 4). F. Schematic illustration of this study. Ach: acetylcholine, AchE: acetylcholinesterase, α7nAchR: nicotinic acetylcholine receptor alpha 7, BLM: bleomycin, Ctrl: control, DON: donepezil, IL17A: interleukin 17A, PH: pulmonary hypertension, Th17: T helper 17. JAK2: Janus kinase 2, STAT3: signal transducer and activator of transcription 3. ^#^*P* < 0.05, *versus* BLM group.

To evaluate the changes in the cholinergic system after DON treatment, AchE activity in plasma and lung tissue was measured. DON significantly reduced AchE activity, by 20.95% and 39.39%, in the plasma and lung tissue of BLM-induced mice, respectively (all *P* < 0.05, Figure 7A-B). This indicates that DON effectively enhanced the parasympathetic activity by inhibiting AchE activity *in vivo*.

In our earlier work, DON was shown to exert an anti-inflammatory effect via α7nAchR in a PH model. Additionally, we proposed that DON may function as a direct activator for the JAK2-STAT3 pathway (Figure S10). Therefore, we further determined the relative level of the cholinergic receptor α7nAchR in lung tissue in the BLM-induced mouse model. Consistently, the level of α7nAchR in the lung tissue of BLM-induced mice is significantly decreased after DON treatment (*P* < 0.05, Figure 7C). Moreover, the level of α7nAchR in Th17 cells were also detected. Immunofluorescence staining showed α7nAchR is abundant in Th17 cells, which could be reduced by DON treatment (Figure 7D). Western blot quantitative analysis also revealed that the level of α7nAchR in Th17 cells was downregulated after DON intervention, however, without a statistical difference (Figure 7E). These results suggest that DON effectively enhances parasympathetic activity and its function in regulating Th17 cell differentiation depends on α7nAchR.

## Discussion

This study showed that DON improved pulmonary artery and RV remodeling in a BLM-induced mouse model. The RNA-seq results indicated that DON mainly exerted anti-inflammatory and anti-fibrotic functions in this process. In humans and in the animal model, Th17 cells significantly infiltrated lung tissue during the fibrotic process, activating lung fibroblasts by secreting IL17A. Nevertheless, DON increased parasympathetic activation, which manifested as inhibition of AchE activity and α7nAchR expression. Based on which, the differentiation of Th17 cells was suppressed by blocking the JAK2-STAT3 pathway (Figure 7F).

Parasympathetic activation has been identified to reverse the course of PH 12, 13. da Silva Goncalves Bos et al. 12 tested the potential therapeutic effects of pyridostigmine, another AchE inhibitor, in stimulating parasympathetic activity. They found that enhancing parasympathetic activity effectively improved the survival and RV function and reduced pulmonary vascular remodeling in experimental rats induced by hypoxia/SU5416. Similarly, Yoshida et al. 13 used vagus nerve stimulation (VNS) to restore autonomic balance, in which they observed that parasympathetic activity effectively ameliorated pulmonary vascular remodeling and preserved RV function in an animal model 13. In a previous study, we showed that DON-enhanced parasympathetic activity could effectively reverse the course of pulmonary arterial hypertension in monocrotaline‑induced rats by inhibiting macrophage activation 14. Interestingly, these beneficial effects of parasympathetic activity for PH are associated with Group I PH. In contrast to previous studies, this study further supported a protective effect of DON on Group III PH, which occurs frequently and features intense local and systemic inflammation.

In this study, we observed an anti-fibrotic function of DON in lung fibrosis. The anti-fibrotic function of parasympathetic activity has been reported in various diseases, mainly associated with inhibiting the inflammatory response 19-21. In addition to its protective function in PH, parasympathetic activity is also protective against severe pulmonary diseases 12, 22, 23. In a rat model of acute respiratory distress syndrome, Li et al. 22 found VNS stimulating parasympathetic activity prevented lung injury by decreasing the secretion of pro-inflammatory factors TNFα and IL1β and increasing the level of the anti-inflammatory factor IL10. Tornero et al. 24 further observed the effect of VNS on patients with severe coronavirus disease 2019 (COVID-19): VNS therapy for 5 days significantly reduced inflammatory markers, specifically C-reactive protein and procalcitonin, with satisfactory safety. This suggested that stimulating parasympathetic activity in the early inflammatory stage could effectively prevent the progression of the fibrotic process.

However, the beneficial effect of parasympathetic activity on lung fibrosis remains controversial. Although they share similar inflammatory characteristics, it has been reported that vagotomization of the lungs resulted in less collagen deposition and prevented lung fibrosis, while increased activity of the vagus nerve exerted the opposite effect 25. Herein, we showed that the protective effects of parasympathetic activity exerted by DON differed from previous findings, which might have two explanations. One explanation might result from the early delivery of DON after BLM exposure in mice, which exerts a potent anti-inflammatory function in the early stage and effectively blocks its progression to fibrosis. Moreover, the noncholinergic mechanisms of DON in regulating biological function might be another reason for this difference.

Inflammatory cell infiltration determines the progress of lung diseases. The inflammatory cell profile of lung tissue has been analyzed in lung fibrosis and PH, and a cluster of immune cells, including monocytes, macrophages, mast cells, neutrophils, dendritic cells, and B and T lymphocytes, have been found to exert essential functions in this disease process 15, 16. After cell distribution analysis, a recent study further reported that T lymphocytes and their subtypes were closely related to collagen deposition in lung fibrosis after a BLM challenge, revealing the importance of T lymphocytes in promoting the fibrotic process 26. Takei et al. 27 reported activation of aryl hydrocarbon receptor signals in lung fibrosis and changes in immunological features, mainly presented as increased Tregs and suppression of inflammatory T cell subsets. Birnhuber et al. 28 reported that the pro-fibrotic actions derived from the immune system were unbalanced and that blockade of IL1 could worsen lung function by increasing Th2 inflammation and collagen production. Besides, the pro-fibrotic effect of Th17 cells was also identified in lung fibrosis 29, 30. In this study, we tested the function of Th17 cells in the progression of lung fibrosis. While *in vitro* studies utilizing Th17 cells co-cultured with fibroblasts provided insights into their function, an analysis of sorted Th17 cells from lung tissue might more accurately delineate their role in fibroblast activation. This may represent a limitation of the present study. Overall, we showed that this process could be significantly inhibited by DON treatment, suggesting a promising therapeutic target for lung fibrosis-induced PH.

We observed that Th17 cells stimulate lung fibroblasts to modulate extracellular matrix deposition by secreting IL17A. IL17A is a typical pro-fibrotic factor and could be released by Th17 cells 31. A previous study reported that both normal and pathogenic lung fibroblasts expressed functional IL17RA, which could respond to IL17A stimulation to generate extracellular matrix proteins, suggesting that the IL17A-regulated pathway exerts essential functions in idiopathic pulmonary fibrosis and rheumatoid arthritis-associated lung disease 32. Using antibody agents that block the function of IL17 in clinical use, IL17 has been identified as a compelling antiphlogistic driver for inflammatory disease 33. However, the effect of IL17A on diseases remains somewhat controversial: the pro-proliferative role of IL17A has been shown to promote recovery after injury 33. In this study, we have also tested that IL17A recombinant protein exerts fewer effects on fibroblast activation than Th17 cells. This might suggest that IL17A derived from specific cells in different pathological conditions exerts distinctive functions, and Th17-derived IL17A possesses potent pro-fibrotic properties.

Parasympathetic activation mainly exerts its biological function by releasing the neurotransmitter acetylcholine, which generally binds to its nicotinic and muscarinic receptors. However, acetylcholine could be degraded rapidly by AchE, which suggested that both an AchE inhibitor and its related receptor could be potential action sites for parasympathetic activation. Recently, Vang et al. 34 found that α7nAChR activation led to an increase in adult ventricular fibroblast proliferation and collagen content, and inhibition of α7nAChR is a potentially novel therapeutic strategy in the setting of increased RV afterload in a PH model. Previously, we observed that parasympathetic activity protected against PH by downregulating α7nAchR expression 14. Similarly, in this study, the AchE and α7nAchR levels were also assayed to verify the underlying mechanism of DON in BLM-induced mice, and α7nAchR was considered a key factor in the regulation of Th17 cell differentiation. It is noteworthy that the observed downregulation of α7nAChR in our study could potentially be attributed to feedback resulting from an increase in acetylcholine levels. As such, our findings highlight the necessity for further research to gain a better understanding of the intricate mechanisms involved in this process.

JAK2 is a non-receptor tyrosine kinase activated by a broad spectrum of stimulators, which has been reported as the most predominant subform activated in lung fibrosis 35-37. Evidence shows that the activation of JAK2 could effectively increase the level of phosphorylated STAT3 and promote its polymerization into dimers, thereby regulating gene transcription 35. Zhang et al. 37 reported that blockade of JAK2/STAT3 activity could suppress hypoxia-induced pulmonary artery smooth muscle cell proliferation, both *in vitro* and *in vivo*, which contributes to inhibiting pulmonary vascular remodeling in PH. Celada et al. 38 reported that inhibiting STAT3 expression in T lymphocytes could mediate the release of TGFβ and IL17A, thereby ameliorating fibroblast activation and lung fibrosis. In contrast to previous findings, our results further supported the involvement of the α7nAChR-mediated JAK2/STAT3 pathway in the differentiation of Th17 cells, revealing a specific action of this pathway in regulating lung fibrosis.

Overall, we attempted to explore the protective effect of parasympathetic activation in Group III PH using a BLM-induced mouse model characterized by severe inflammation and fibrosis in lung tissue 26, 39, 40. BLM is a cytotoxic agent used to treat a variety of neoplasms, and its cytotoxicity occurs mainly in the lung 41. After BLM treatment, the lung undergoes significant biochemical, histological, and physiological changes that provide helpful insights into the mechanisms of lung injury, repair, and fibrosis 41. Interestingly, the remodeling of muscular pulmonary arteries and arterioles appears to be more or less restricted to the fibrotic lung areas in this model, resulting in pulmonary artery remodeling that is not proportional to the severity of pulmonary lesions. Moreover, lung fibrosis might occur with or without an inflammatory response in the clinic, leading to limited therapeutic function in some patients after immunosuppressive therapy 42. In this study, our findings established the biological significance of parasympathetic activation, α7nAchR, Th17 cells, and IL17A as potential therapeutic targets for patients suffering from lung fibrosis-induced PH. However, further studies are still needed to confirm these effects.

## Conclusion

PH secondary to lung fibrosis is classified as Group III PH, which is common and associated with a worse prognosis. The determining factor of lung fibrosis is fibroblast activation and excessive extracellular matrix deposition; however, no treatment can reverse this course. In this study, our results supported the view that the AchE inhibitor DON could effectively ameliorate pulmonary artery and RV remodeling and improve the survival rate in the BLM-induced mouse model. Specifically, DON was shown to repress Th17 cell differentiation and infiltration in lung tissue to prevent the progression of lung fibrosis. Besides, DON regulation of Th17 cell differentiation depends on the α7nAchR-JAK2-STAT3 pathway. Our findings provide more evidence that DON ameliorates pulmonary inflammation and offers a potential therapeutic strategy to treat lung fibrosis-related PH.

## Supplementary Material

Supplementary methods, figures and tables.

## Abbreviations

α7nAchR: nicotinic acetylcholine receptor alpha 7
αSMA: alpha-smooth muscle actin
AchE: acetylcholinesterase
BLM: bleomycin
DON: Donepezil
DEGs: differentially expressed genes
GO: Gene ontology
GEO: Gene Expression Omnibus
ILs: interleukins
IL17RA: IL17A receptor
LV: left ventricle
PH: Pulmonary hypertension
PPI: protein-protein interaction
RV: right ventricle
RVSP: RV systolic pressure
SEM: standard error of the mean
Th17: helper 17
Treg: regulatory T
VNS: vagus nerve stimulation
WT: wall thickness
