# Supplementary material for: Inhibition of Th17 cells by donepezil ameliorates experimental lung fibrosis and pulmonary hypertension
Source: Theranostics. 2023 Mar 21;13(6):1826–42. doi: 10.7150/thno.82069 (PMC10091879; doi:10.7150/thno.82069)
Supplement: Supplementary file 1 — Supplementary methods, figures and tables. [file thnov13p1826s1.pdf]

Supplementary Material:

Inhibition of Th17 cells by donepezil ameliorates experimental lung fibrosis and pulmonary hypertension

Yuan Guo[1, 2], Ziyu He[2], Zhu Chen[3], Fengling Chen[2], Chengming Wang[2], Wanlu Zhou[1], Jie Liu[1], Hao Liu[1], Ruizheng Shi[1]*

[1.] Department of Cardiovascular Medicine, Xiangya Hospital, Central South University, Changsha 410008, Hunan, China.

[2.] Department of Cardiovascular Medicine, Zhuzhou Hospital Affiliated to Xiangya School of Medicine, Central South University, Zhuzhou 412007, Hunan, China.

[3.] Hunan Key Laboratory of Biomedical Nanomaterials and Devices, Hunan University of Technology, Zhuzhou 412007, Hunan, China.

Corresponding Author:

Ruizheng Shi, Department of Cardiovascular Medicine, Xiangya Hospital, Central South University, No. 87 Xiangya Road, Changsha 410008, Hunan, China. Tel: +86 13755082530, E-mail: xyshiruizheng@csu.edu.cn.

Short title: Donepezil ameliorates lung fibrosis induced PH

1. Materials and Methods

1.1 Measurement of echocardiography

Echocardiography was used to evaluate the function of right ventricle (RV) at the end of the experiment. Echocardiographic evaluation was measured by transthoracic echocardiography using a 22 MHz phased array transducer (VINNO8, China). The indicators of pulmonary arterial acceleration time (PAAT), pulmonary ejection time (PET), and tricuspid annular plane systolic excursion (TAPSE) were measured in the parasternal short axis and apical four chamber heart view respectively [1, 2].

1.2 Assessment of hemodynamics

The mice were anesthetized with 1% pentobarbital, and RV systolic pressure (RVSP) was measured using a 1.2-French microtip pressure transducer catheter. After carefully inserted into the RV, RVSP was continuously recorded and analyzed by RM6240E instrument (Chengdu instrument factory, China).

1.3 Histology of lung and heart

Lung tissue and heart of the mice were harvested, and respectively stained with Hematoxylin and eosin (HE) and Masson's trichrome. Lumen obstruction and wall thickness (WT) rate were measured to reflect pulmonary arterial remodeling. The right ventricle hypertrophy index (RVHI) was calculated to confirm RV remodeling, which was represented as the weight of RV free wall to the left ventricle (LV) and ventricular septum (S) (RVHI=RV / (LV + S)) [3].

1.4 RNA sequencing (RNA-seq) analysis

Lung tissue in each group were used for RNA-seq analysis. Total RNA was extracted with TRIzol reagent (Invitrogen, USA). Complementary DNA library construction was performed and DNA nanoballs (DNBs) were loaded into the patterned nanoarray and single end 50 bases reads were generated on BGIseq500 platform (BGI-Shenzhen, China). The sequencing data was filtered with SOAPnuke (v1.5.2) and clean reads were obtained and stored in FASTQ format. The clean reads were mapped to the reference genome using HISAT2 (v2.0.4). Essentially, clean read counts were loaded into *BGI Dr. Tom* platform and analyzed with DESeq2 (v1.4.5). Genes with adjusted values of $P < 0.05$ and $|\log2FC| > 2.0$ were deemed to be differentially expressed genes (DEGs).

1.5 Flow cytometric analysis

Flow cytometry was used to identify Th17 cells, which was defined as highly expressed CD3, CD4, and interleukin (IL) 17A. Cells were diluted to 2×10^{6}/100 μL and incubated with a non-specific binding blocking reagent cocktail of purified anti-mouse CD16/CD32 antibody in cell staining buffer (BioLegend, USA). Cell surface antigens were stained with the fluorochrome-conjugated antibodies of APC-anti-mouse CD4 (BioLegend, USA; 0.25 μg/μL), and FITC-anti-mouse CD3 (BioLegend, USA; 1 μg/μL). After fixation and permeabilization, intranuclear factors were stained with fluorochrome-conjugated antibodies of PE-anti-mouse IL17A (BioLegend, USA; 0.25 μg/μL). The data were acquired on a flow cytometer (Cytek Aurora, USA) and analyzed

using FlowJo (Treestar, USA) software.

1.6 T lymphocyte isolation and Th17 cell differentiation

Naïve CD4 positive T lymphocytes were isolated from mouse spleen with EasySep™ Mouse CD4 Positive Selection Kit II (Stemcell Technologies, Canada) according to the manufacturer's protocol. After verifying the sorting efficiency, cells were used immediately to induce Th17 cell differentiation.

Differentiation of naïve CD4 positive T cells into Th17 cells was induced by CellXVivo Mouse Th17 Cell Differentiation Kit (R&D Systems, USA) according to the manufacturer's instruction. After 5 days cultivation, IL17A expression was used to evaluate the differentiation rate of Th17 cells.

1.7 Immunohistochemistry staining

Lung tissues were stained with CD68 and CD3 to quantify inflammatory cell infiltration. Briefly, rabbit anti-CD68 and anti-CD3 antibodies (1:500 dilution, Abcam, USA) were used and immunohistochemical staining was performed using the two-step immunohistochemical technique with diaminobenzidine (DAB, Sigma-Aldrich, USA) following the manufacturer's instructions. The positive stained cells were counted by image J software (NIH, Bethesda, MD, USA), and presented as the mean of five randomly selected fields from scanned images (DSAssistantLite software, Motic, China; ×400 magnification, image size: 715×408 μm). CD68 and CD3 positive rate were calculated as integral optical density (IOD)/area in each field.

1.8 Immunofluorescence staining

Immunofluorescence staining for lung tissue, lung fibroblasts and Th17 cells was respectively performed according to the manufacturer's protocol of Immunol Fluorescence Staining Kit (Beyotime Biotechnology, China). Briefly, lung tissue was stained with alpha-smooth muscle actin (αSMA) specific mouse monoclonal antibody (1:300 dilution, Proteintech, China). Lung fibroblasts were stained with αSMA specific mouse monoclonal antibody (1:300 dilution, Proteintech, China), anti-collagen I rabbit antibody (1:200 dilution, Servicebio, China), and vimentin rabbit monoclonal antibody (1:200 dilution, Beyotime Biotechnology, China). Th17 cells was stained with rabbit anti-nicotinic acetylcholine receptor alpha7 (α7nAchR) antibody (1:200 dilution, Abcam, USA). After incubated overnight at 4°C, cells were incubated with Alexa Fluor 555-Labeled Donkey Anti-Rabbit IgG or FITC-Labeled Goat Anti-Mouse IgG conjugated secondary antibody. Cells were counterstained with DAPI (Beyotime Biotechnology, China) at the end of the immunostaining. Fluorescent positive stained cells were detected with an Olympus CKX53 inverted research microscope.

1.9 Mouse inflammation antibody array detection

To explore the activation and function of Th17 cells, we employed a mouse inflammation antibody array-membrane (ab133999, Abcam, USA) to detect inflammatory factors and cytokines in the Th17 cell differentiation medium. The detection protocol was carried out in accordance with the kit instructions, and a comprehensive panel of 40 mouse inflammatory factors and cytokines was assayed.

1.10 ELISA test

Th17 cell culture medium and the medium from Th17 cell co-cultured with lung fibroblasts were centrifuged at 500 g for 10 min to remove the remaining cellular components. The supernatant was obtained for ELISA detection. The concentration of inflammatory factor IL17A was detected using ELISA kit (ABclonal Technology, China) following the manufacturer's protocol.

Acetylcholinesterase (AchE) activity in plasma and lung tissue in each group were respectively measured by colorimetry according to the manufacturer's protocol (Acetylcholinesterase assay kit, Nanjingjiancheng Bioengineering Institute, China) [3].

1.11 Western blotting

Protein obtained from lung tissue and cells were determined according to a standard protocol [4]. Western blotting was performed with rabbit polyclonal α7nAchR antibody (1:500 dilution, Abcam, USA), rabbit collagen I antibody (1:1000 dilution, Servicebio, China), rabbit collagen III antibody (1:1000 dilution, Servicebio, China), rabbit phospho-JAK2-Y1007/1008 polyclonal antibody (1:1000 dilution, ABclonal, China), rabbit anti-JAK2 polyclonal antibody (1:1000 dilution, Servicebio, China), rabbit anti-STAT3 (phosphorY705) antibody (1:6000 dilution, Abcam, USA), rabbit anti-STAT3 polyclonal antibody (1:1000 dilution, Servicebio, China), αSMA specific mouse monoclonal antibody (1:5000 dilution, Proteintech, China), anti-IL17A receptor rabbit polyclonal antibody (1:1000 dilution, Servicebio, China), anti-βactin rabbit polyclonal (1:1000 dilution, Servicebio, China), goat anti-rabbit (HRP) IgG antibody (1:5000 dilution, ZSGB-Bio, China), and goat anti-mouse (HRP) IgG antibody (1:5000

dilution, ZSGB-Bio, China). ImageJ (NIH, Bethesda, MD, USA) was used to quantify the pixel intensities of immunoreactive bands.

1.12 Real-time quantitative PCR (RT-PCR)

Total RNA was extracted from the lung tissue of mice in each group with RNAeasy™ Animal RNA Isolation Kit with Spin Column (Beyotime Biotechnology, China). One microgram of RNA was used for cDNA-synthesis by RevertAid First Strand cDNA Synthesis Kit (Thermofisher, USA). RT-PCR was performed using TaqMan™ assay probes and detected by Applied Biosystems 7500 instrument. The sequences of the primer pairs used in this study were listed as follows. Data were normalized to Gapdh and expressed as a relative ratio.

Genes		Sequences
Il17a	Forward	TTTAACTCCCTTGGCGCAAAA
	Reverse	CTTTCCCTCCGCATTGACAC
Col1a1	Forward	GCTCCTCTTAGGGGCCACT
	Reverse	CCACGTCTCACCATTGGGG
Timp1	Forward	GCAACTCGGACCTGGTCATAA
	Reverse	CGGCCCGTGATGAGAAACT
Serpinb2	Forward	GTGCTGGGGGTAACACTGAAC
	Reverse	GCGAAATCACAGCCACTGAAG
Ccl2	Forward	TTAAAAACCTGGATCGGAACCAA
	Reverse	GCATTAGCTTCAGATTTACGGGT
	Forward	ATTGGTTGGAGAGGAAGCGG

Tbx21	Reverse	GCACCAGGTTCGTGACTGTA
Gapdh	Forward	TGGCCTTCCGTGTTCCTAC
	Reverse	GAGTTGCTGTTGAAGTCGCA

References

1. Zhu Z, Godana D, Li A, Rodriguez B, Gu C, Tang H, et al. Echocardiographic assessment of right ventricular function in experimental pulmonary hypertension. Pulm Circ. 2019; 9: 2045894019841987.

2. Kohut A, Patel N, Singh H. Comprehensive Echocardiographic Assessment of the Right Ventricle in Murine Models. J Cardiovasc Ultrasound. 2016; 24: 229-38.

3. Qiu H, Zhang Y, Li Z, Jiang P, Guo S, He Y, et al. Donepezil Ameliorates Pulmonary Arterial Hypertension by Inhibiting M2-Macrophage Activation. Front Cardiovasc Med. 2021; 8: 639541.

4. Guo Y, Luo F, Zhang X, Chen J, Shen L, Zhu Y, et al. TPPU enhanced exercise-induced epoxyeicosatrienoic acid concentrations to exert cardioprotection in mice after myocardial infarction. J Cell Mol Med. 2018; 22: 1489-500.

2. Tables

Table S1: Details of microarray datasets information

GEO ID	Platform	Tissue	Attributing participants	Organism
GSE24988	GPL6244 [HuGene-1_0-st] Affymetrix Human Gene 1.0 ST Array [transcript (gene) version]	Lung	non-PH=22; PH=62	Homo sapiens
GSE15197	GPL6480 Agilent-014850 Whole Human Genome Microarray 4x44K G4112F (Probe Name version)	Lung	non-PH=13; PH=8	Homo sapiens
GSE48149	GPL16221 Illumina HumanRef-8 v3.0 expression beadchip (Search Key version)	Lung	non-PH=9; PH=8	Homo sapiens

Table 2: 159 DEGs in lung tissue of BLM-induced mice after DON treated

Gene ID	Gene Symbol	Type	Log 2 (DON/BLM)	Log 2 (BLM/Ctrl)	P value (DON/BLM)	P value (BLM/Ctrl)	Q value (DON/BLM)	Q value (BLM/Ctrl)
100034684	'Cstdc5'	mRNA	-6.55298	4.671733	5.73E-06	3.64E-05	0.002346	0.001471
100044068	'LOC100044068'	mRNA	-2.3813	3.49996	0.168729	0.093556	0.938587	0.362799
100504404	'H2-Ea-ps'	mRNA	11.37307	-10.5326	1.24E-38	9.99E-10	1.14E-34	2.60E-07
105244804	'Gm40349'	mRNA	-2.10592	2.387398	0.127981	0.184257	0.903254	0.525091
108167918	'Gm46290'	mRNA	5.132033	-5.38946	0.002676	0.045076	0.173879	0.23808
108168003	'Gm46353'	mRNA	-6.16273	6.175172	0.0091	0.012359	0.34293	0.105711
108169105	'LOC108169105'	mRNA	-4.80914	4.823911	0.073883	0.088074	0.811858	0.350375
110308	'Krt5'	mRNA	-2.93864	2.998416	0.201171	0.24239	0.965144	0.601721
11535	'Adm'	mRNA	-2.51818	2.759966	1.38E-04	2.99E-05	0.028862	0.001256
11801	'Cd5l'	mRNA	-2.56727	4.714346	0.006055	2.01E-05	0.266923	9.27E-04
11846	'Arg1'	mRNA	-3.74203	6.071237	0.006234	2.45E-05	0.271566	0.001084
12035	'Bcat1'	mRNA	-3.43786	3.248551	8.31E-04	0.00683	0.09007	0.071803
12310	'Calca'	mRNA	-3.32961	4.156695	0.0015	0.007389	0.121206	0.075808
12424	'Cck'	mRNA	-5.56495	8.014982	7.02E-04	0.011574	0.081419	0.101056
12722	'Clca3a1'	mRNA	-2.42669	3.711328	2.63E-05	1.38E-08	0.008498	2.33E-06
12814	'Col11a1'	mRNA	-3.89143	6.823232	0.011789	4.16E-08	0.397042	5.69E-06
12842	'Col1a1'	mRNA	-2.00982	2.74017	0.001279	1.40E-05	0.111609	6.88E-04
12904	'Crabp2'	mRNA	-3.10166	4.095393	0.010111	0.006329	0.361787	0.068035
13349	'Ackr1'	mRNA	-2.6802	4.879298	2.11E-07	5.07E-18	1.49E-04	1.03E-14
13386	'Dlk1'	mRNA	-3.79671	2.769442	3.04E-05	0.032418	0.009672	0.195004
13447	'Doc2b'	mRNA	-2.2882	2.486857	0.016199	0.007837	0.450578	0.078935
14115	'Fbln2'	mRNA	-2.44933	2.018965	8.49E-04	0.046288	0.09043	0.242063
14276	'Folr2'	mRNA	-2.41824	2.394404	0.003905	0.008363	0.211635	0.082323
14283	'Fosl1'	mRNA	-3.33504	3.830716	0.003095	0.004735	0.188907	0.05562
14417	'Gad2'	mRNA	-2.92852	2.546531	0.011725	0.045577	0.396459	0.239513
14472	'Gbx2'	mRNA	-2.81086	4.705467	0.011829	0.002988	0.397042	0.040491
14763	'Gpr37'	mRNA	-3.93822	3.183822	0.004896	0.034048	0.242536	0.201132
14938	'Gzma'	mRNA	2.257031	-2.67651	8.84E-04	8.29E-05	0.091529	0.002754
15006	'H2-Q1'	mRNA	5.091228	-5.67913	1.43E-05	5.09E-08	0.004975	6.68E-06
15033	'H2-T18'	mRNA	8.224272	-6.69034	0.03536	3.84E-06	0.615818	2.42E-04
15077	'H3c14'	mRNA	6.83947	-6.00485	0.036628	0.108773	0.625473	0.393985
15129	'Hbb-b1'	mRNA	3.740973	-13.444	0.049237	2.94E-05	0.697951	0.001251
15218	'Foxn1'	mRNA	-3.32352	5.107869	0.142124	0.069784	0.921194	0.308245
15364	'Hmga2'	mRNA	-4.69053	5.881981	0.002996	2.29E-04	0.18526	0.006081
15551	'Htr1b'	mRNA	-3.16652	2.276937	1.84E-04	0.00585	0.034936	0.064245
15891	'Ibsp'	mRNA	-1.97383	5.475607	0.065543	0.001429	0.782774	0.02384
16178	'Il1r2'	mRNA	-2.82151	3.077596	5.02E-06	7.94E-07	0.002202	6.61E-05
16323	'Inhba'	mRNA	-2.78472	3.652394	3.68E-04	1.45E-05	0.053292	7.04E-04
16447	'Ivl'	mRNA	-3.3531	2.361686	2.30E-04	0.081799	0.042008	0.33636

16635	'Klra4'	mRNA	1.978747	-3.24106	0.066894	0.00182	0.787177	0.028457
16641	'Klrc1'	mRNA	2.227064	-2.34054	0.008519	0.003224	0.328442	0.042781
16663	'Krt13'	mRNA	-6.28246	4.438856	0.002007	0.019025	0.143898	0.140247
16664	'Krt14'	mRNA	-3.25253	6.147711	0.039793	0.003168	0.638795	0.042189
16666	'Krt16'	mRNA	-2.98534	5.763967	0.086355	0.004407	0.836674	0.05285
16687	'Krt6a'	mRNA	-3.03755	7.863953	0.017336	1.25E-06	0.469803	9.66E-05
16688	'Krt6b'	mRNA	-5.57079	5.582985	0.004078	0.005118	0.21785	0.058732
16948	'Lox'	mRNA	-2.16746	2.280386	6.01E-06	2.23E-06	0.002408	1.54E-04
170677	'Cdhr1'	mRNA	-2.04509	3.430221	0.077998	0.026286	0.82559	0.17144
17181	'Matn2'	mRNA	-2.20614	2.539207	4.73E-04	1.60E-04	0.063127	0.004649
17384	'Mmp10'	mRNA	-2.36603	6.149396	0.038201	2.98E-04	0.635758	0.007464
17393	'Mmp7'	mRNA	-4.88475	4.899066	0.068525	0.082057	0.792704	0.336686
17394	'Mmp8'	mRNA	-2.49213	3.544179	8.00E-15	3.49E-23	2.95E-11	1.06E-19
17750	'Mt2'	mRNA	-2.15227	3.591037	0.022566	3.11E-04	0.523081	0.007634
17897	'Myl3'	mRNA	2.486074	-4.48442	0.282087	0.215392	0.988263	0.567385
18167	'Npy2r'	mRNA	-2.0447	3.693566	0.117051	0.022821	0.891493	0.156499
18416	'Otc'	mRNA	2.751172	-2.68642	0.023954	0.035451	0.535712	0.20661
18491	'Pappa'	mRNA	-4.03089	4.38706	3.51E-05	9.28E-06	0.010603	4.93E-04
18772	'Pkp1'	mRNA	-2.53259	5.987795	0.068102	6.95E-04	0.79206	0.013895
18788	'Serpinb2'	mRNA	-2.23455	3.125759	0.021054	0.002519	0.507837	0.035955
19242	'Ptn'	mRNA	-1.99904	2.266397	0.02828	0.015181	0.57673	0.121114
19416	'Rasd1'	mRNA	-2.33566	2.699887	2.27E-06	1.24E-04	0.001128	0.003784
195209	'Zfp469'	mRNA	-2.15418	2.906244	0.002403	1.11E-04	0.162145	0.003503
19699	'Reln'	mRNA	-2.04549	2.120753	3.70E-04	3.53E-04	0.053292	0.008304
19737	'Rgs5'	mRNA	-3.33717	2.880732	6.77E-08	3.23E-06	5.67E-05	2.09E-04
20198	'S100a4'	mRNA	-2.35244	3.42206	0.001904	8.72E-06	0.139813	4.72E-04
20201	'S100a8'	mRNA	-2.65996	2.187454	2.37E-07	5.38E-05	1.62E-04	0.00201
20210	'Saa3'	mRNA	-2.28007	5.5528	0.003846	1.64E-11	0.209719	6.24E-09
20254	'Scg2'	mRNA	-2.83449	2.493284	0.018396	0.082431	0.480861	0.337887
20296	'Ccl2'	mRNA	-2.57993	5.345762	0.00493	4.77E-08	0.24355	6.30E-06
20306	'Ccl7'	mRNA	-2.83037	4.887857	0.001352	1.38E-06	0.113737	1.05E-04
20307	'Ccl8'	mRNA	-1.93417	3.089504	0.009773	2.45E-04	0.355424	0.006392
20617	'Snca'	mRNA	3.114878	-2.40526	1.86E-04	0.001544	0.03498	0.025265
20716	'Serpina3n'	mRNA	-2.66393	3.805264	3.24E-05	3.24E-09	0.009942	6.95E-07
20717	'Serpina3m'	mRNA	-3.31775	4.219449	1.30E-04	1.10E-06	0.028495	8.69E-05
20862	'Stfa2'	mRNA	-7.63284	6.683412	3.11E-07	6.10E-06	1.97E-04	3.54E-04
21418	'Tfap2a'	mRNA	-2.09484	3.615632	0.020294	2.07E-04	0.50116	0.005655
214305	'Hhipl1'	mRNA	-2.89424	2.436568	0.001588	0.009692	0.123442	0.090609
21673	'Dntt'	mRNA	-3.99122	3.371215	0.012354	0.055387	0.404587	0.269976
216739	'Acsl6'	mRNA	-2.5686	3.558211	0.013677	0.001756	0.422708	0.027746
217212	'Pyy'	mRNA	-4.38527	6.261496	0.006	0.001365	0.266021	0.0231
217951	'Tmem196'	mRNA	-2.82269	2.674993	0.010241	0.025044	0.365721	0.166201
21826	'Thbs2'	mRNA	-2.24745	3.059454	5.51E-04	7.78E-06	0.069511	4.36E-04

21857	'Timp1'	mRNA	-2.41299	4.34192	6.58E-04	1.71E-09	0.078256	3.86E-07
22409	'Wnt10a'	mRNA	-2.56641	6.512567	0.014381	7.87E-08	0.424892	9.50E-06
22412	'Wnt9b'	mRNA	-2.74517	3.956982	0.019939	0.001645	0.50059	0.026546
22420	'Wnt6'	mRNA	-2.11121	2.762664	0.048732	0.018373	0.696692	0.137438
225266	'Klhl14'	mRNA	2.276342	-2.73508	0.110874	0.020241	0.881877	0.145671
227358	'Erfe'	mRNA	-2.10205	3.597118	0.005203	1.17E-05	0.250103	5.86E-04
227632	'Kcnt1'	mRNA	-4.37815	5.969424	1.02E-05	1.68E-07	0.003834	1.80E-05
228770	'Rspo4'	mRNA	2.58057	-3.1545	9.65E-05	2.25E-04	0.022803	0.00601
228785	'Mylk2'	mRNA	-3.43367	3.418406	0.01589	0.038646	0.44749	0.217171
230777	'Hcrtr1'	mRNA	-1.96183	3.31805	0.141604	0.034255	0.921194	0.201852
231293	'Cwh43'	mRNA	-2.92729	6.796243	0.009195	1.68E-04	0.34512	0.004846
235505	'Cd109'	mRNA	-3.01878	4.346521	0.009623	3.83E-04	0.351834	0.008852
235712	'Mrgpra2b'	mRNA	-3.60845	3.138494	0.001061	0.001963	0.101329	0.029996
237560	'Lrrc10'	mRNA	-2.1251	-2.48927	0.061289	0.15313	0.76104	0.474565
23892	'Grem1'	mRNA	-3.74777	5.082831	0.005449	3.13E-04	0.255486	0.00767
239853	'Adgrg7'	mRNA	-3.43502	4.885317	0.014031	0.004472	0.423243	0.053393
240873	'Tnfsf18'	mRNA	-1.9952	6.63142	0.003109	2.15E-06	0.189061	1.50E-04
245195	'Retnlg'	mRNA	-2.53779	2.756851	8.62E-09	3.18E-06	1.06E-05	2.06E-04
258407	'Olfr464'	mRNA	-2.51535	3.646964	0.153659	0.024678	0.933171	0.164791
26367	'Ceacam2'	mRNA	-6.39855	7.420953	0.008264	0.050292	0.324001	0.254676
26388	'Ifi202b'	mRNA	-3.06363	2.889554	2.63E-04	0.03158	0.044535	0.19222
268885	'Stfa2l1'	mRNA	-5.24632	4.197745	5.93E-15	1.11E-11	2.73E-11	4.60E-09
269116	'Nfasc'	mRNA	-1.98012	3.547941	0.030362	0.011233	0.58863	0.09922
271127	'Adamts16'	mRNA	-5.15655	5.16975	0.057947	0.06951	0.745146	0.307764
27206	'Nrk'	mRNA	-3.74302	4.026136	0.001228	6.42E-04	0.110418	0.013099
277353	'Tcfl5'	mRNA	-2.18034	5.665239	0.05914	0.002305	0.751901	0.033768
278180	'Vsig4'	mRNA	-3.74932	3.732673	0.001659	0.002232	0.127846	0.032986
319433	'Serpine3'	mRNA	-4.2802	3.702527	0.024752	0.030153	0.543954	0.186824
319942	'A530016L24Rik'	mRNA	3.501784	-4.93586	0.063315	1.88E-04	0.769766	0.005291
320772	'Mdga2'	mRNA	-2.32294	4.523589	0.123737	0.028306	0.899579	0.179248
328829	'9830107B12Rik'	mRNA	-2.5881	2.560083	0.015225	0.014126	0.436801	0.115738
381413	'Gpr176'	mRNA	-1.98892	4.334141	0.002813	2.64E-08	0.178153	4.02E-06
382864	'Colq'	mRNA	2.245805	-3.63231	0.006439	6.49E-06	0.276605	3.71E-04
433016	'Cstdc4'	mRNA	-7.01578	4.134243	6.58E-07	6.59E-06	3.79E-04	3.76E-04
433182	'Eno1b'	mRNA	-2.04753	2.408531	0.129223	0.077002	0.904509	0.326459
53867	'Col5a3'	mRNA	-2.51721	3.244815	0.001001	3.46E-05	0.098596	0.001411
546644	'Ly6g'	mRNA	-2.11083	2.340345	0.011331	0.007444	0.389111	0.0762
547349	'LOC547349'	mRNA	8.692589	-8.89548	1.74E-07	1.66E-09	1.33E-04	3.84E-07
56857	'Slc37a2'	mRNA	-2.20537	3.571602	1.19E-04	3.28E-09	0.026705	6.95E-07
57765	'Tbx21'	mRNA	2.090784	-2.22754	0.008225	0.004813	0.323881	0.056278
58179	'Klrc3'	mRNA	2.158828	-2.83981	0.004517	1.35E-04	0.231886	0.004023
65115	'Bean1'	mRNA	-2.67774	2.317598	0.004103	0.011528	0.217877	0.100841
654824	'Ankrd37'	mRNA	-2.98838	3.983546	1.61E-04	8.39E-07	0.031559	6.93E-05

666279	'Dspp'	mRNA	-4.77857	7.255197	0.001943	5.55E-04	0.142105	0.011787
666907	'Ms4a4a'	mRNA	-2.62418	2.536324	5.76E-04	0.002245	0.071415	0.033081
67405	'Nts'	mRNA	-2.11934	2.181621	0.077526	0.088926	0.823604	0.352045
67645	'Armc12'	mRNA	-2.72649	2.323124	0.085915	0.08447	0.835497	0.342809
677156	'Cyp4f37'	mRNA	3.424371	-2.85198	2.01E-04	0.150809	0.037111	0.471498
67855	'Asprv1'	mRNA	-2.89494	2.451331	0.00146	6.03E-04	0.120429	0.012581
68588	'Cthrc1'	mRNA	-2.00946	2.803818	0.008915	7.49E-04	0.337351	0.014645
69142	'Cd209f'	mRNA	2.272258	-3.30833	0.009895	0.001213	0.35753	0.021159
69623	'Zfp33b'	mRNA	3.034987	-3.49995	0.009581	9.62E-04	0.351025	0.017582
69717	'Gm10499'	mRNA	6.746969	-5.42232	3.18E-50	1.77E-08	5.85E-46	2.85E-06
70417	'Megf10'	mRNA	-2.40958	3.513972	0.034065	0.001421	0.61041	0.023764
70835	'Prss22'	mRNA	-2.17116	5.742134	0.024873	1.71E-05	0.543954	8.06E-04
71584	'Gdpd2'	mRNA	-2.38609	2.411713	0.002437	0.003848	0.163196	0.047921
71781	'Slc16a14'	mRNA	-2.20316	2.332624	8.20E-04	0.001794	0.09007	0.028168
71797	'Chst13'	mRNA	-4.30492	3.424102	0.010989	0.041013	0.383516	0.224815
71854	'Dpep3'	mRNA	-2.16394	2.645611	0.034885	0.013088	0.614008	0.110033
71912	'Jsrp1'	mRNA	-3.31633	5.22862	0.089495	0.058663	0.840611	0.278196
72077	'Gcnt3'	mRNA	-4.74551	3.799404	0.021803	0.044807	0.51577	0.236966
72780	'Rspo3'	mRNA	-2.36982	2.907647	0.003465	6.11E-04	0.203263	0.012695
74145	'F13a1'	mRNA	-2.80205	2.82853	2.43E-05	9.10E-06	0.007994	4.85E-04
74175	'Crct1'	mRNA	-2.66071	6.28558	0.084539	0.010719	0.833805	0.096406
74463	'Exoc3l2'	mRNA	-2.15193	2.183642	0.001536	0.00202	0.121458	0.030745
74488	'Lrrc15'	mRNA	-3.55858	6.427194	8.57E-04	4.06E-07	0.09043	3.68E-05
75497	'Fabp12'	mRNA	3.245953	-4.35792	0.002139	1.51E-06	0.15104	1.12E-04
75552	'Paqr9'	mRNA	2.188729	-2.79059	0.013947	0.010826	0.422708	0.097079
76229	'Vmn2r29'	mRNA	4.701752	-5.26477	0.091695	0.003707	0.842159	0.047033
78354	'2210407C18Rik'	mRNA	-2.52313	2.893781	0.202842	0.226218	0.965144	0.58178
80797	'Clca3a2'	mRNA	-2.3107	5.405173	0.005133	8.68E-06	0.248901	4.71E-04
80982	'Cemip'	mRNA	-2.68284	4.318131	8.29E-04	7.97E-08	0.09007	9.56E-06
85031	'Pla1a'	mRNA	-2.31966	3.316771	8.69E-04	1.76E-05	0.09043	8.25E-04
93835	'Amn'	mRNA	-3.06412	2.715377	0.029262	0.053031	0.580447	0.26212
94047	'Tmem121b'	mRNA	2.138939	-3.39166	0.037977	6.19E-05	0.635648	0.002193
94226	'S1pr5'	mRNA	2.960527	-3.00087	8.35E-04	8.44E-04	0.09007	0.015842
96875	'Prg4'	mRNA	-2.23729	3.277886	0.006901	1.83E-04	0.293023	0.005175

BLM: bleomycin, Ctrl: control, DON: donepezil, DEGs: differentially expressed genes.

Table S3. Inflammatory factors and cytokines change during Th17 cell differentiation. The culture medium of both naïve T lymphocytes and Th17 cells were assayed using a mouse inflammation antibody array-membrane. A total of 40 mouse inflammatory factors and cytokines were measured and the results were quantified as grayscale values. Out of these, 12 exhibited a greater than twofold change.

	Grayscale values											
	GMCSF	IL2	IL7	IL6	IL12 p40/p70	IL17	Leptin	TECK	MIG	TIMP1	sTNF RI	sTNF R II
Naïve T	0.155	0.117	0.060	0.012	0.009	0.059	0.024	0.209	0.013	0.089	0.102	0.244
Th17	0.463	1.347	0.898	0.823	0.957	1.573	0.001	0.095	0.005	0.340	1.516	0.786

3. Figures

Figure S1. Analysis flowchart of the microarray datasets

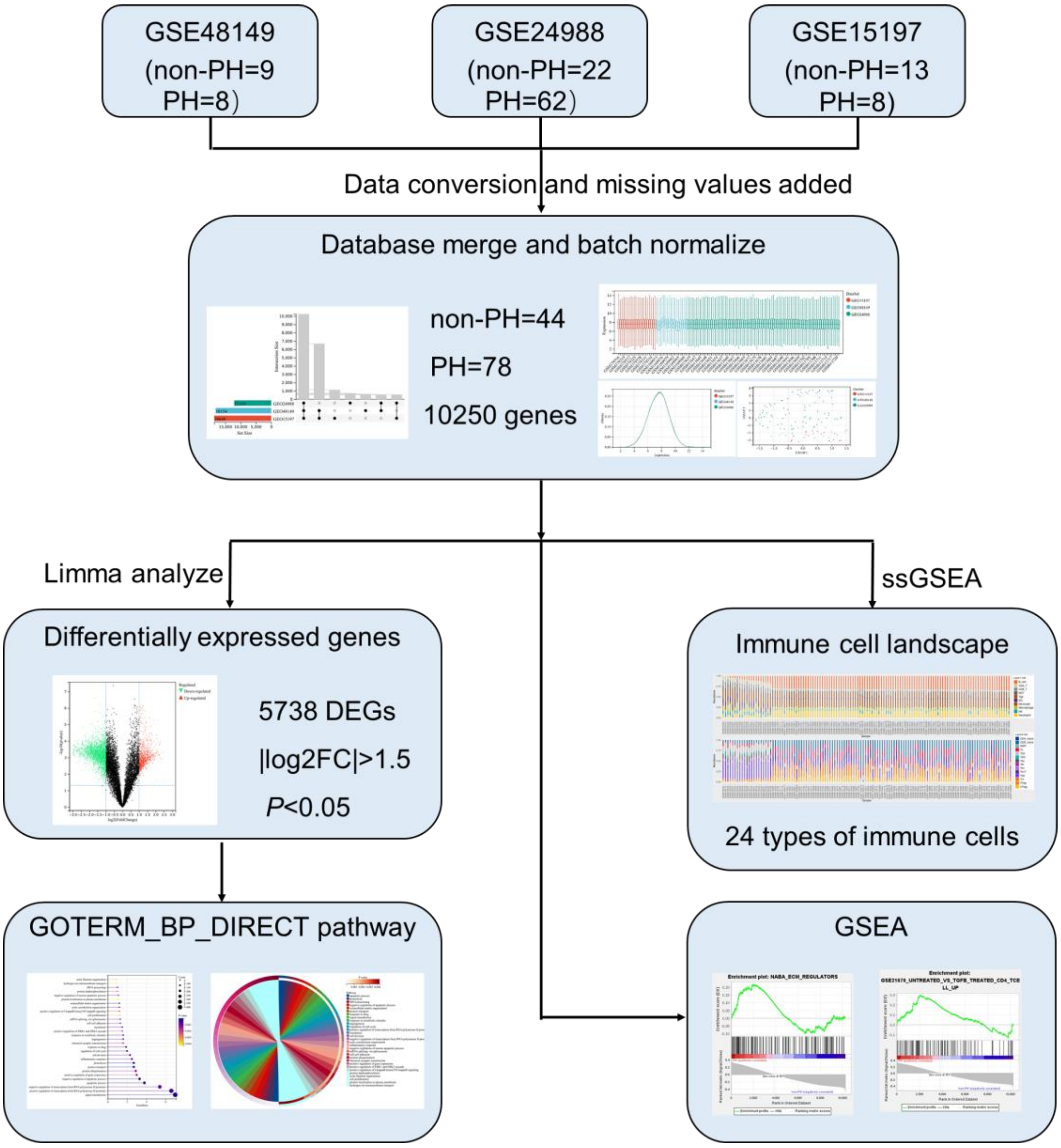

Figure S2. Microarray datasets merge and performance of batch normalize. A. Merging of datasets. 10250 genes were co-expressed in the three datasets of GSE24988, GSE48149, and GSE15197. B. Prior plots of batch fitting effect. C. Boxplot of expression intensity of each sample in datasets after normalization. D. UMAP plot after batch-adjustment.

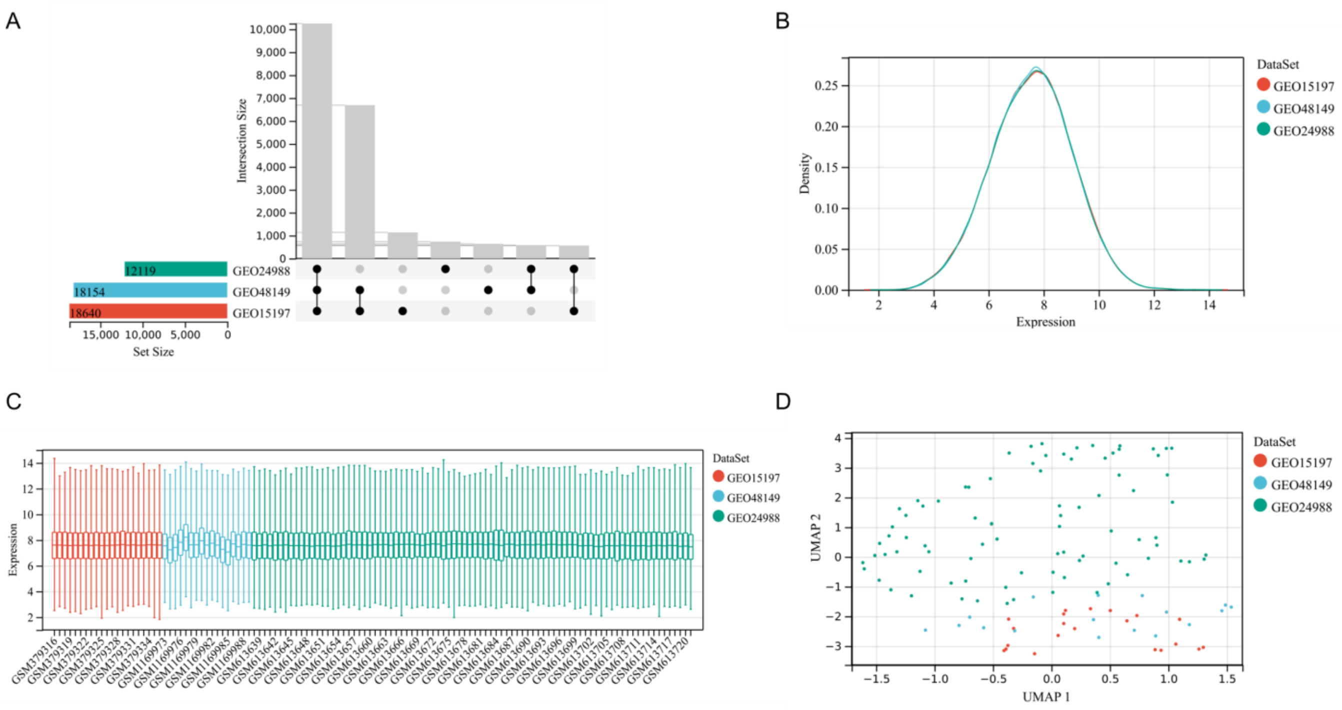

Figure S3. Identification of lung fibroblasts. Lung fibroblasts were isolated and cultured *in vitro*. After six days of cultivation, immunofluorescence staining with vimentin was performed, and the positive stained cells were defined as lung fibroblasts. A. Immunofluorescence staining (×400 magnification). B. Immunofluorescence staining (×200 magnification). C. Immunofluorescence staining (×100 magnification).

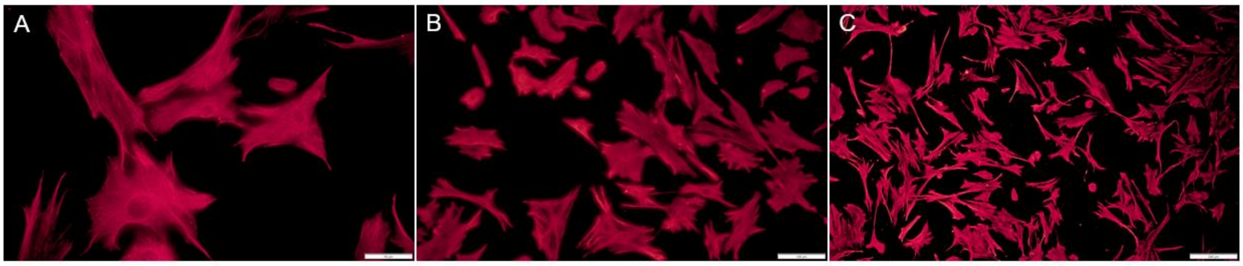

Figure S4. Heat map of the most closely interreacted 40 DEGs screened by PPI. BLM: bleomycin, Ctrl: control, DON: donepezil, DEGs: differentially expressed genes, PPI: protein-protein interaction.

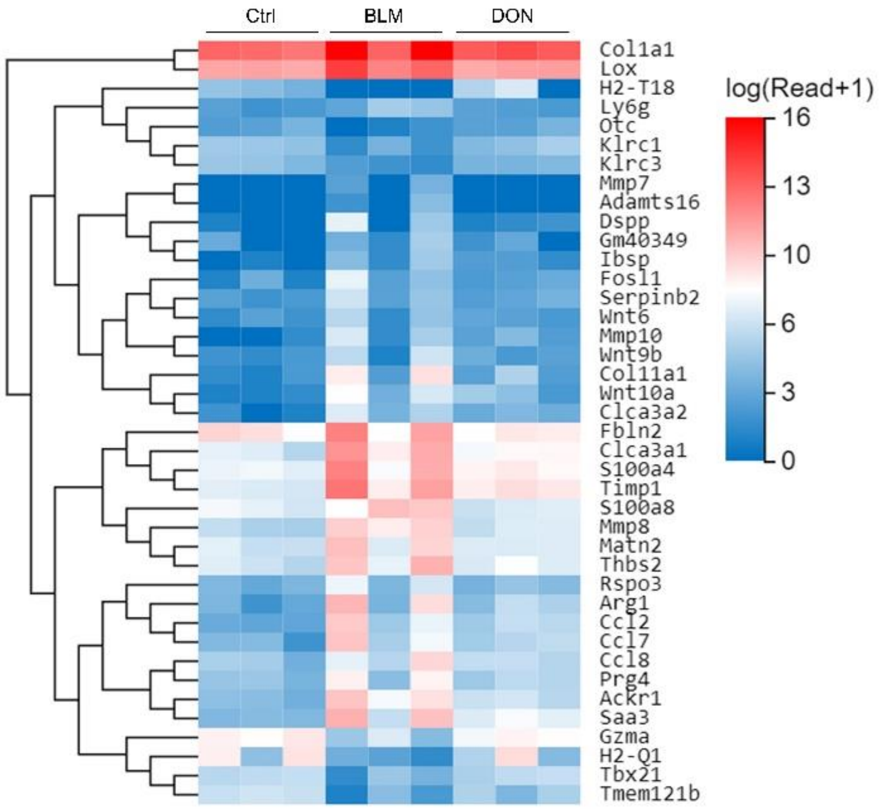

Figure S5. Heat map showing the relationship between 24 different types of immune cells infiltrating in lung tissue between patients with PH and non-PH controls. PH: pulmonary hypertension. Red: positive correlation; white: the same correlation levels; and blue: negative correlation.

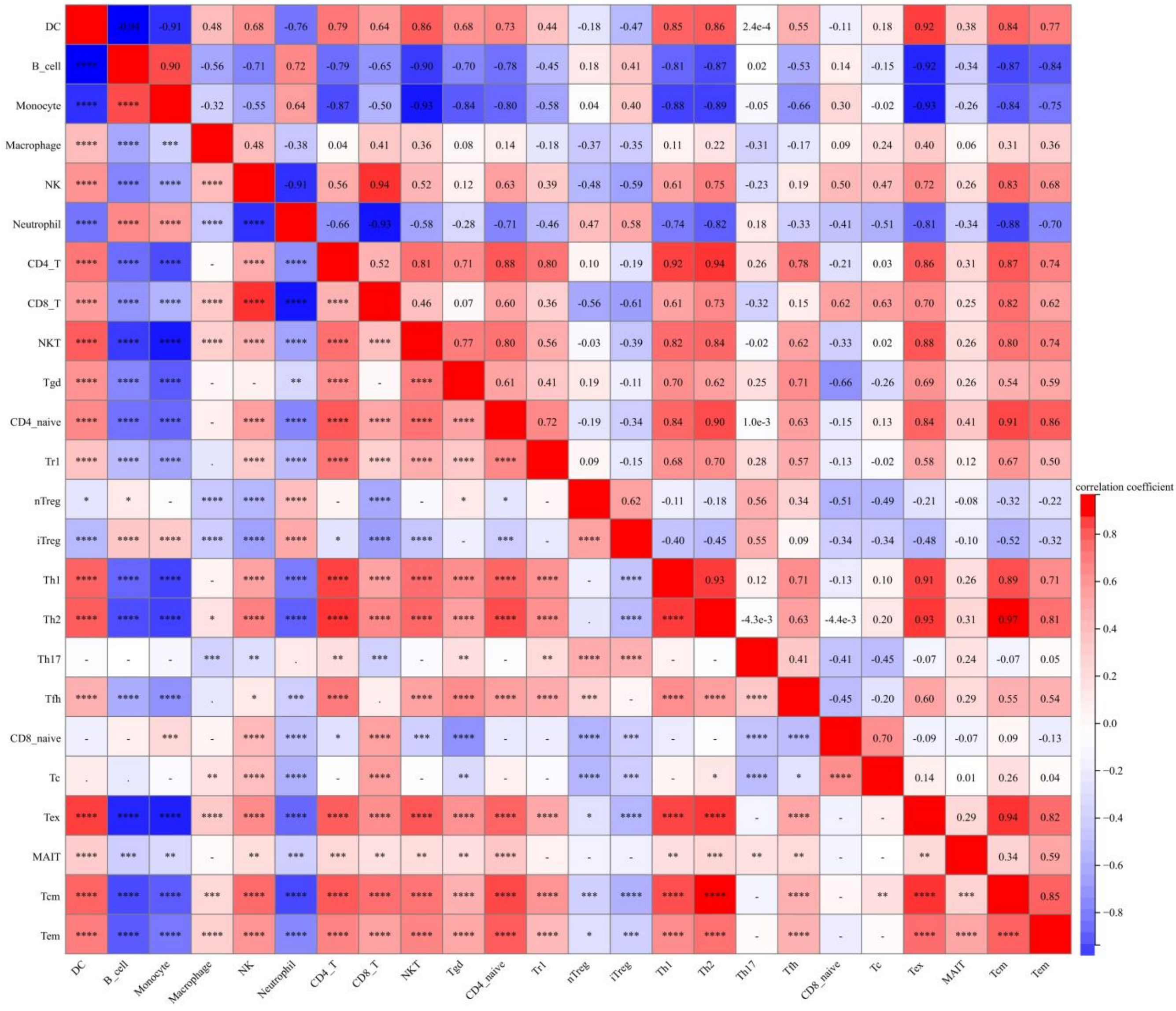

Figure S6. Least absolute shrinkage and selection operator (LASSO) logistic regression was used to screen the signature of Th17 cells in PH process. A. Establishment of signatures by LASSO logistic regression analysis. LASSO coefficient profile of the 17 genes. B. Selection of the optimal parameter (lambda) in the LASSO model, and generation of a coefficient profile plot. C. LASSO logistic regression screening the infiltration of Th17 cell in lung tissue of patients with PH secondary to lung fibrosis and non-PH controls.

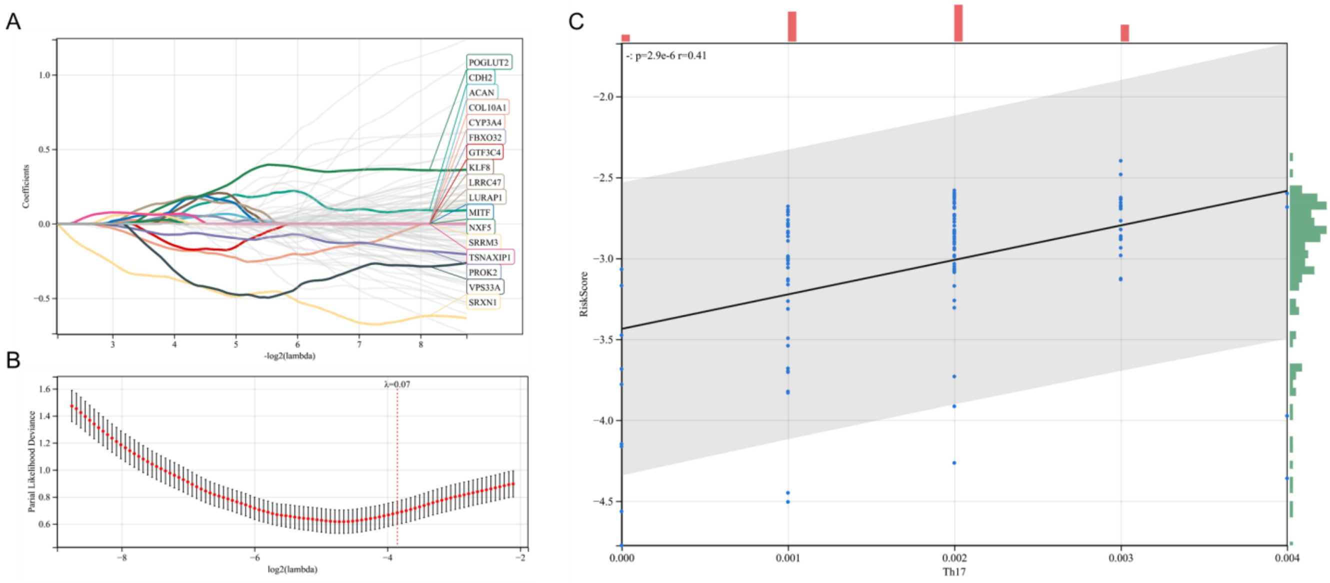

Figure S7. Flow cytometry gating strategy of analyzing Th17 cells. $CD3^{+}CD4^{+}IL17A^{+}$ cells were defined as Th17 cells. IL17A: interleukin 17A, Th17: T helper 17.

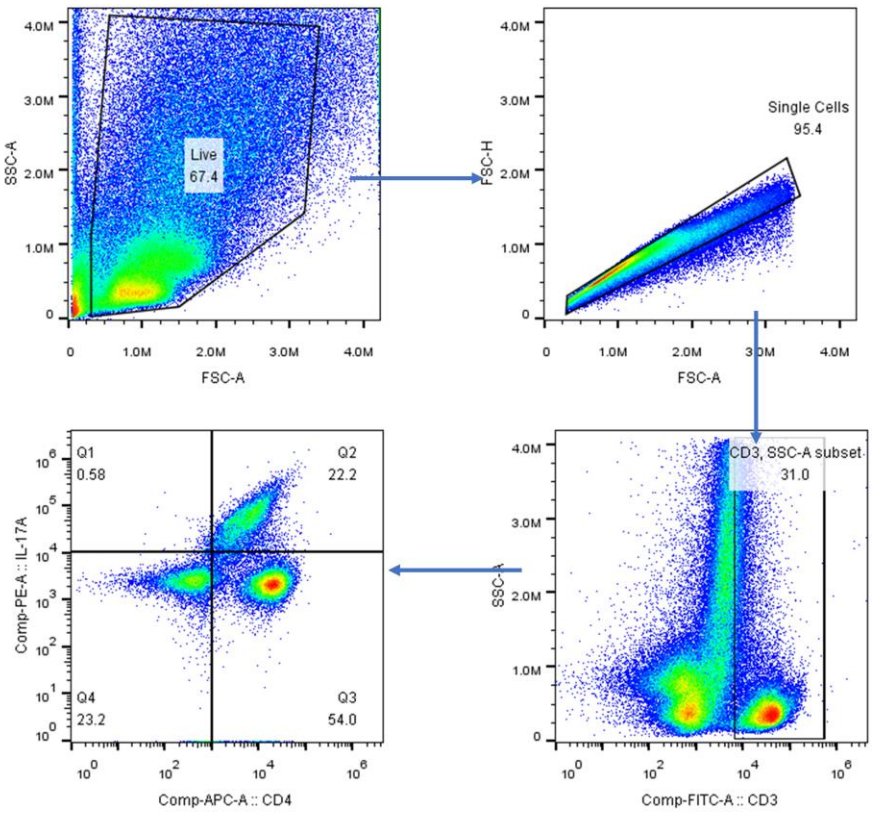

Figure S8. Cell morphology of naïve T lymphocytes and Th17 cells. The upper row of pictures shows naïve T lymphocytes, and the lower row shows Th17 cells that cultured at 5 days. A~B. Magnification ×400, C~D. Magnification ×200, E~F. Magnification ×100. Th17: T helper 17.

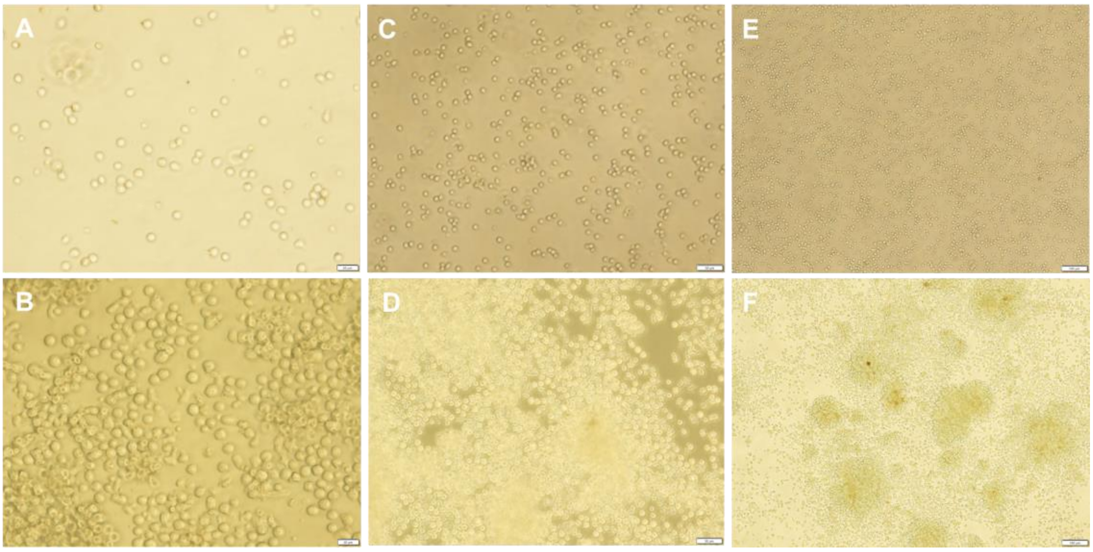

Figure S9. The activation of fibroblasts that treated by IL17A. To detect the effect of IL17A on fibroblasts, 1 μg/mL anti-IL17A (R&D Systems, USA) neutralizing antibody was used to incubate with fibroblasts, and then 0.1 μg/mL IL17A recombinant protein (ABclonal, China) were added to fibroblasts with or without IL17A. After another 48 hours cultivation, the relative expression of collagen I, collagen III and αSMA was identified by western blot. A. Representative protein levels in lung tissue. B~D. Statistical analysis of the levels of collagen I, collagen III, and αSMA in lung tissue (n = 4~5).

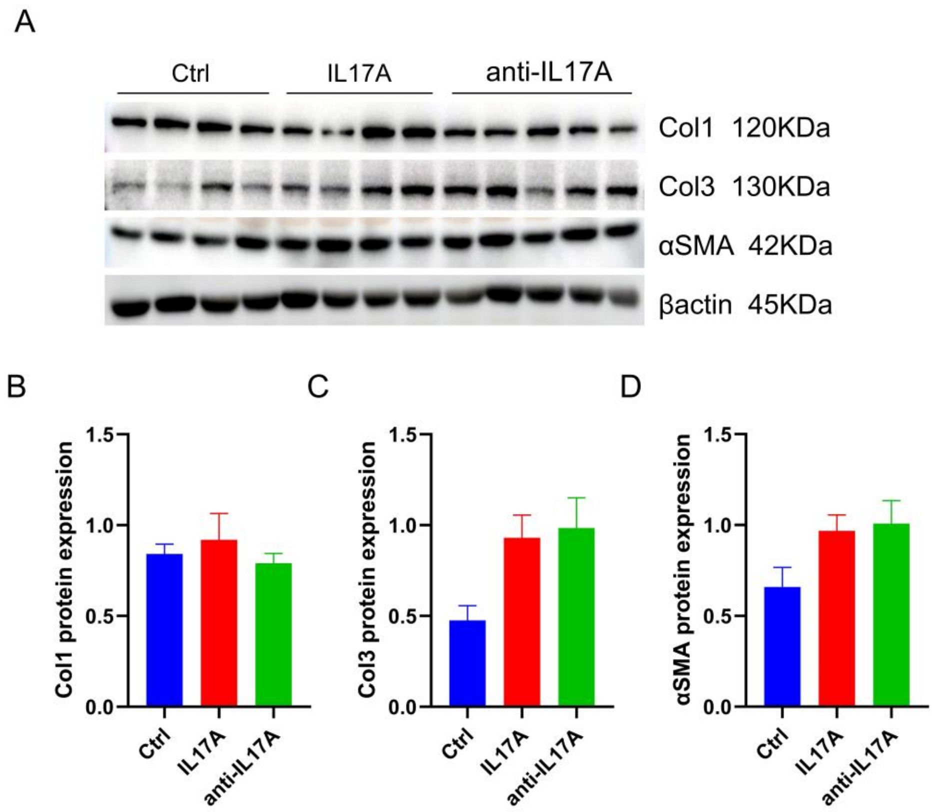

Figure S10. The JAK2 signaling pathway was identified as being highly interrelated to α7nAChR. To further elucidate the biological effects of α7nAChR, we utilized the *String database* to predict the optimal signaling pathway. Our analysis revealed that the JAK2 pathway scored the highest among all the predicted pathways.

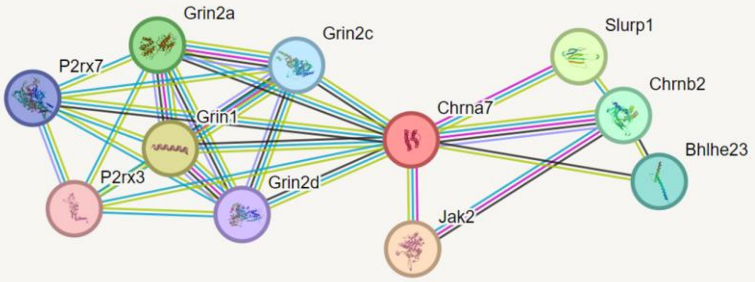

Your Input:

Chrna7	*Cholinergic receptor, nicotinic, alpha polypeptide 7; Neuronal acetylcholine receptor subunit alpha-7; After binding acetylcholine, the AChR responds by an extensive change in conformation that affects all subunits and leads to opening of an ion-conducting channel across the plasma membrane. The channel is blocked by alpha-bungarotoxin (502 aa)*

Predicted Functional Partners:

		Neighborhood	Gene Fusion	Cooccurence	Coexpression	Experiments	Databases	Textmining	[Homology]	Score
Jak2	*Janus kinase 2; Non-receptor tyrosine kinase involved in various processes such as cell growth, development, differentiat...*					●	●	●		0.995
Grin1	*Glutamate receptor, ionotropic, nmda1 (zeta 1); Glutamate receptor ionotropic, NMDA 1; Component of NMDA receptor c...*				●		●	●		0.976
Slurp1	*Secreted Ly-6/uPAR-related protein 1; Has an antitumor activity. Was found to be a marker of late differentiation of the sk...*					●	●	●		0.951
Grin2a	*Glutamate receptor ionotropic, NMDA 2A; Component of NMDA receptor complexes that function as heterotetrameric, lig...*				●		●	●		0.950
Chrnb2	*Cholinergic receptor, nicotinic, beta polypeptide 2 (neuronal); Neuronal acetylcholine receptor subunit beta-2; After bindin...*				●	●	●	●	●	0.919
Bhlhe23	*Class E basic helix-loop-helix protein 23; May function as transcriptional repressor. May modulate the expression of gene...*				●			●		0.861
Grin2c	*Glutamate receptor ionotropic, NMDA 2C; Component of NMDA receptor complexes that function as heterotetrameric, lig...*				●		●	●		0.856
P2rx7	*Purinergic receptor p2x, ligand-gated ion channel, 7; P2X purinoceptor 7; Receptor for ATP that acts as a ligand-gated ion...*				●		●	●		0.852
Grin2d	*Glutamate receptor ionotropic, NMDA 2D; Component of NMDA receptor complexes that function as heterotetrameric, lig...*				●		●	●		0.844
P2rx3	*P2X purinoceptor 3; Receptor for ATP that acts as a ligand-gated ion channel; Belongs to the P2X receptor family*						●	●		0.835
